# Infrared Technology for Vein Visualization: Device Development

**DOI:** 10.3390/bioengineering13101217

**Published:** 2026-10-19

**Authors:** Nicholas V. Ivanov, Anca-Iulia Nicu, Nicolae Marius Roman

**Affiliations:** Department of Electrical Engineering and Measurement, Faculty of Electrical Engineering, Technical University of Cluj-Napoca, 400114 Cluj-Napoca, Romania; anca.nicu@ethm.utcluj.ro (A.-I.N.);

**Keywords:** NIR, veins, segmentation, filter, image processing

## Abstract

Difficult peripheral venous access can complicate routine venipuncture, especially when superficial veins are poorly visible under normal lighting conditions. This study aims to develop and preliminarily evaluate a low-cost near-infrared imaging prototype for non-invasive visualization of superficial veins. The prototype consists of a camera without an infrared light filter, an infrared LED illumination module, and an image-processing pipeline implemented in MATLAB. Acquired images were processed using median filtering, histogram-based contrast enhancement, binarization, inversion, and masking procedures to improve vein visibility and outline vascular structures. The prototype was tested on three participants, with three anatomical regions examined for each participant, resulting in nine analyzed images. Vein-to-background contrast and contrast-to-noise ratio were calculated before and after image processing. Across the analyzed images, mean contrast increased from 0.14968 to 0.29088, with a mean relative improvement of 113.40%, while mean CNR increased from 0.48179 to 1.00729, with a mean relative improvement of 130.65%. These results indicate that the proposed system improved the objective visibility of superficial veins under controlled acquisition conditions. The study provides an initial technical basis for further development, while further studies are required to evaluate its performance in larger and more diverse participant groups.

## 1. Introduction

For administering intravenous therapy or collecting venous blood samples, venipuncture is a necessary procedure, which can be disrupted for various reasons. Therefore, a vein locator device is a useful tool not only to make venipuncture safe and accurate but also to reduce the patient’s tension and anxiety and to increase the efficiency and comfort of donation or sample collection. Near-Infrared (NIR) imaging technology is widely used in the biomedical field. By using NIR imaging, it is possible to map normal and aberrant veins and may assist in procedures involving patients whose veins are difficult to visualize. The present work was designed as an initial technical feasibility study to develop a low-cost near-infrared imaging prototype and to evaluate whether a reproducible MATLAB-based image-processing pipeline could improve objective image-quality indicators for superficial vein visualization. The technical hypothesis was that the proposed acquisition and processing workflow would increase vein-to-background contrast and contrast-to-noise ratio in NIR images acquired under controlled conditions [1,2,3].

### 1.1. Research Objectives

Vein visualization technology uses non-invasive infrared technology to capture and obtain an enhanced image of superficial veins, even in the current offline acquisition and processing workflow, with real-time implementation considered as a future development objective. The sample skin is exposed to near-infrared (NIR) light from an array of 36,960 nm 12 V LEDs during the procedure performed by this equipment. The illuminated area is then imaged using a Raspberry Pi NoIR camera module, sourced from Optimus Digital, Bucharest, Romania.

The acquired images are transferred to a computer, where they are processed and filtered before visualization on the monitor. Volunteers of different ages and genders were included in the preliminary evaluation. Overall, the experimental results indicate that the veins and surrounding tissues on the wrist of the samples can be distinguished by changes in contrast and brightness. The contribution of this work is not the invention of a new segmentation algorithm but the development and preliminary evaluation of a low-cost NIR-based prototype that combines accessible hardware with a reproducible image-processing pipeline for enhancing superficial vein visibility. The study did not aim to assess venipuncture success, diagnostic performance, anatomical segmentation accuracy, or generalizability across different skin phototypes, vascular disorders, or clinically difficult venous access populations.

### 1.2. Analysis of Existing Solutions

There are a variety of vein location technologies available for use in the medical field to locate veins. These devices are designed to help medical personnel locate veins for treatments such as venipuncture (blood sampling), intravenous cannulation, and other medical procedures. Here are some of the most common vein location technologies:

Table 1 compares representative vein-localization technologies according to imaging modality, hardware configuration, processing strategy, strengths, limitations, and their relationship to the proposed low-cost NIR/IR camera-based vein visualization system. As the proposed system is defined by its use of a Raspberry Pi 4 Model B, a Raspberry Pi Camera Module 2 NoIR, and a 36-LED, 960 nm 12 V infrared illumination module. Image processing is performed offline using a MATLAB processing pipeline. Its primary objective is to provide some intel on the development and preliminary evaluation of a low-cost NIR-based prototype. A major distinction between the proposed prototype and high-end commercial systems, such as AccuVein or VeinViewer, is its economic accessibility. While commercial NIR devices are often limited in many settings due to costs ranging from €1500 to €20,000, where the proposed system is constructed from off−the−shelf components with a total parts cost of approximately €191 to €290. This allows technology to be utilized more widely in primary healthcare centers and nursing education programs where expensive proprietary devices are unavailable [3,4,5].

When compared to ultrasound technology, the proposed system differs fundamentally in its imaging modality and ease of use. Ultrasound relies on sound waves to determine the exact depth and diameter of veins, but it requires considerable expertise to operate effectively during a simultaneous cannulation. In contrast, the proposed system uses an operator-independent optical approach, leveraging the fact that deoxygenated hemoglobin absorbs NIR light significantly more than surrounding tissue to create a clear, real-time map on a screen or skin surface [3,6,7].

Finally, the proposed system differs from visible light transilluminators and laser-based detectors in both spectrum and safety profile. Unlike transilluminators that commonly use visible red or orange light and rely on direct visual observation, the proposed prototype uses the invisible near-infrared spectrum and a camera sensor to digitally enhance image contrast. Furthermore, while laser-based systems can pose safety risks such as retinal burns, the proposed device utilizes low-power IR LEDs, would make it a safer alternative for frequent use in clinical environments [3,8].

## 2. Theoretical Notions

### 2.1. Circulatory System

The cardiovascular (CV) system, also known as the circulatory system, is responsible for distributing blood throughout the body and consists of the heart and blood vessels (arteries, capillaries, and veins). An artery is a blood vessel that carries blood from the heart before branching into smaller vessels. Ultimately, the smallest arteries, vessels called arterioles, branch into small capillaries, where nutrients and waste products are exchanged, and then combine with other vessels emerging from the capillaries to form venules, small blood vessels that carry blood to a vein, a larger blood vessel that returns blood to the heart [9,10].

The systemic circulation and the pulmonary circulation are two different ways in which arteries and veins transport blood. Systemic arteries deliver oxygen-rich blood to the body’s tissues. Because much of the oxygen carried by the arteries has been given to the cells, the blood returning to the heart through the systemic veins has less oxygen. In the pulmonary circulation, the arteries carry only low-oxygen blood to the lungs for gas exchange. The heart then pumps the freshly oxygenated blood from the lungs back into the systemic circulation through the pulmonary veins. Although arteries and veins differ physically and functionally, they share several characteristics [9].

In addition to supplying oxygen and other nutrients, such as glucose and amino acids, the circulatory system collects waste products from the body’s cells. Carbon dioxide and other waste products created by metabolic reactions are delivered by the circulatory system to the lungs, liver, and kidneys, where they are eliminated from the body [10].

#### 2.1.1. Venipuncture

Venipuncture is a clinical process that involves drawing blood from a vein using a needle, usually for laboratory testing. Venipuncture can also be used to treat blood problems by removing extra red blood cells from the blood. It is also referred as blood sampling and phlebotomy [7].

#### 2.1.2. Vein Map and Venous Insufficiency

**Figure 1:**
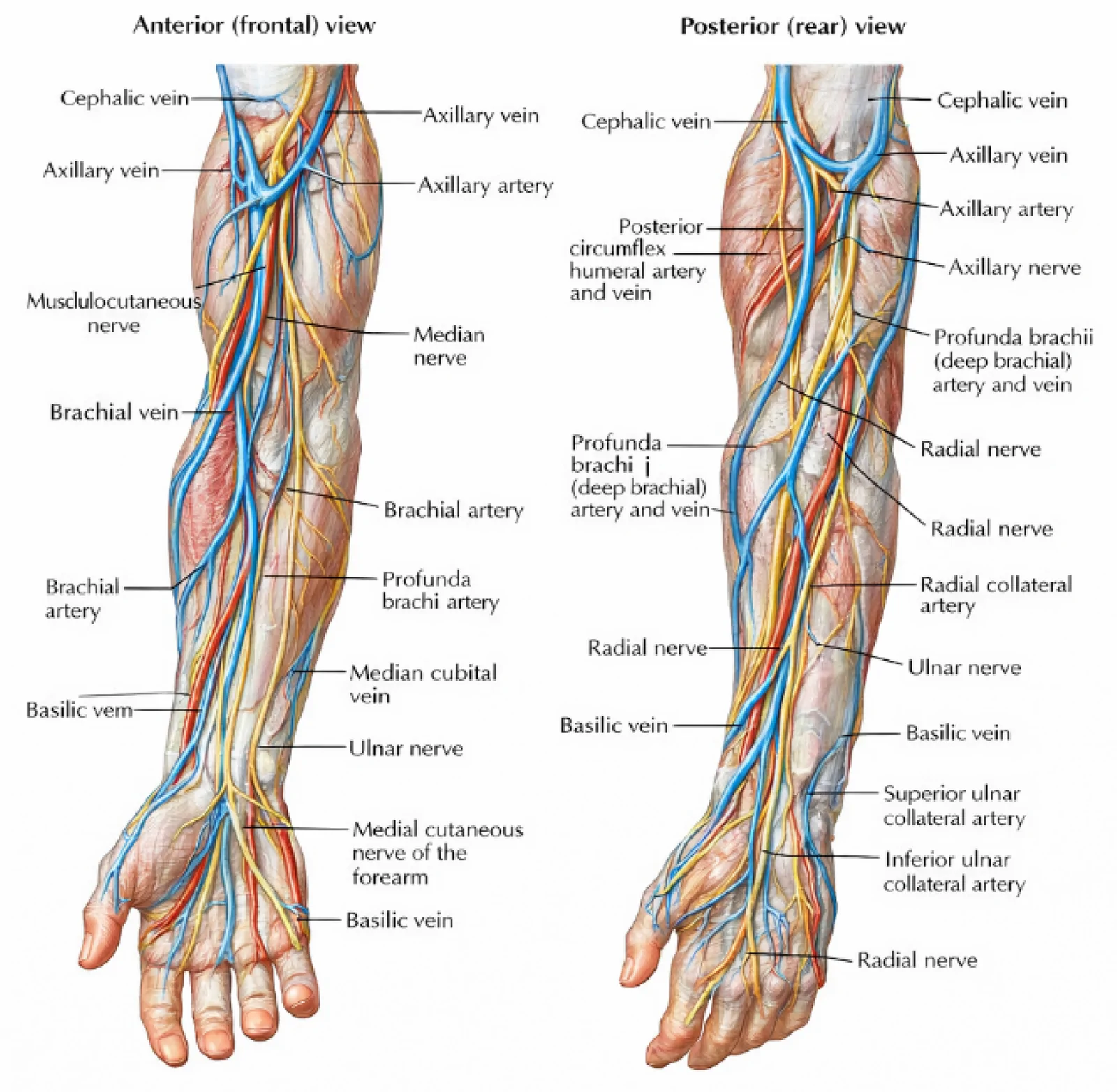
AI-generated schematic representation of the superficial venous anatomy of the upper limb and dorsal hand. Major superficial veins relevant to venipuncture are labelled to indicate their anatomical locations, including the dorsal venous network and dorsal metacarpal veins. The illustration was generated using ChatGPT (GPT-5.2, OpenAI) for anatomical visualization purposes and does not represent patient-derived data.

Venipuncture is most commonly performed on the upper limbs. The antecubital fossa, or the inside of the elbow joint, contains the best veins for venipuncture. The median cubital and cephalic veins are commonly accessible in the antecubital fossa. Due to its proximity to the brachial artery and median nerve, the basilic vein should only be considered if no other vein is more visible. The dorsal aspect of the hand contains the dorsal metacarpal veins and the dorsal venous network and may be used as an alternative site for venipuncture when suitable veins in the antecubital region are unavailable. Venipuncture at this site may be more uncomfortable because the veins are generally smaller and more mobile than those in the antecubital fossa, Figure 1. Venipuncture at this site is more painful because the veins are smaller and more fragile. Using ChatGPT (GPT-5.2), created by OpenAI, artificial intelligence support was given. The program was used to create a visual representation of the venous anatomy of the human arm. The prompt directed the system to create a new image based on multiple publicly available reference images of arm veins, with the requirement that venous structures be clearly highlighted on the arm surface and each major vein explicitly labelled to indicate their anatomical locations, with the scope of simply visualizing the vein structure of the area that are researched in this study. The resulting image is clearly identified as AI-generated in the figure legend [11].

Specimens collected through a hematoma may lead to incorrect test results. Hematomas of any size should not be subjected to phlebotomy. If no other venous site is accessible, the sample should be collected distal to the hematoma. These sites can become thrombosed and damaged as a result of repeated venipunctures and intravenous (IV) injections. As a result, when palpated, the veins in this location may feel “cord-like” or lack definition. In this case, another location should be chosen [11].

#### 2.1.3. Puncture Accuracy

For healthcare professionals who collect blood, visualizing veins is crucial. These procedures may be performed by paramedics, doctors, nurses, and phlebotomists [7,11].

Sometimes, healthcare professionals can quickly identify a vein and draw blood samples. In practice, there are too many cases where inefficient vein identification equipment is at play, leading to failed blood tests or wasted time. Vein finding devices can display images of visible superficial veins to aid in puncture accuracy. High-quality vein visualization tools will also highlight the locations of blood flow in a patient’s veins and display the surrounding tissue. Another benefit of vein visualization devices for phlebotomists is determining a patient’s vein assessment tool (VAT) and successfully donating blood. Using a vein viewer, a patient’s VAT was assessed on a scale of 0 to 12 [7].

The least adequate score was zero, and the most adequate was twelve. The VAT score after vein examination consisted of:

1. Palpation
2. Visibility
3. Size

Patients were not allowed to donate blood if their initial score was less than eight. A poor score would have increased the possibility of re-injection and made it more difficult to donate a sufficient amount of blood [7].

Phlebotomists had a success rate of approximately 90% with patients who scored higher than eight. The median patient score was nine, and when the VAT score was used, phlebotomy success rates were nearly four times higher. Missed target yields and puncture reinjection are the dangers of not using the VAT category. For outpatient clinics specializing in blood donation, a vein detector can be an excellent tool because it helps increase puncture rates and eliminate unsuitable donors [7].

### 2.2. NIR Technology

“The near-IR region is usually considered to include wavelengths between 700 nm (near the red end of the visible spectrum) and 3000 nm (near the beginning of the infrared band of organic compounds). Absorption peaks in the near-IR region come from overtones and combinations of fundamental bands (mid-IR) and from electronic transitions of the heaviest atoms” [12].

Both electronic and vibrational transitions are addressed by NIR spectroscopy. However, distinguishing the NIR region from the visible region is difficult or almost meaningless with electronic spectroscopy. In electronic spectra, the two regions are seamlessly connected. Nevertheless, it is relatively simple to distinguish between vibrational spectroscopy in the NIR region and that in the IR region, since NIR spectroscopy deals only with bands originating from overtones and combination modes, while IR spectroscopy primarily involves bands due to fundamentals, although those originating from supertones and combinations also appear relatively weakly in the IR region [13].

#### 2.2.1. NIR Band

One of the most characteristic features of NIR spectroscopy stems from the fact that the bands in the NIR region are weak or very weak. Both the bands due to electronic transitions and those arising from vibrational transitions are weak. Supertonal modes and combination modes arise from so-called forbidden transitions. The reason why the NIR region is valuable from an application point of view is that only the NIR region offers a highly transmissive window to radiation [13].

The characteristics of the bands that appear in the NIR region can be summarized as follows. Here, we consider NIR vibrational bands, overtones, and combinations:

1. All bands observed in the MR region are the result of overtones and combinations; these include not only simple combinations such as, but also second- and third-order combinations such as. The NIR region has numerous overlapping bands, and MR bands exhibit high multicollinearity. Therefore, it is usually difficult to assign MR bands.
2. Since bands due to higher-order overtones and second- and third-order combinations appear in the shorter wavelength region, NIR bands become increasingly weaker as the wavelength decreases. The wavelength, wave number, and relative intensity of the bands resulting from the fundamental, first, second, and third overtones of the CH stretching mode of chloroform are tabulated in Table 2. It can be seen that the third overtone band is found in the Vis region and that the overtone bands weaken dramatically as the order increases.
3. Most of the bands in the NIR region originate from functional groups (such as OH, CH, and NH) containing a hydrogen atom. This is partly due to the huge enharmonic constant of an XH bond and partly due to the fact that the fundamental frequency of an XH stretching vibration (3800–2800 cm^−1^) is high. NIR spectroscopy is so often referred to as “an XH spectroscopic method.” The second overtone of the C = O stretching modes, in addition to the XH vibration bands, also manifests itself in the NIR region. Recently, bands resulting from the first and second C = N stretching modes of acetonitrile have also been observed.
4. As with the equivalent fundamental bands in IR spectra, the first overtones of the XH stretching bands exhibit a smaller frequency shift when establishing a hydrogen bond or inter- or intramolecular contact. The first overtones shift approximately twice as much as the corresponding IR bands.
5. Compared to the IR region, the OH and NH stretching bands of monomeric and polymeric species can be distinguished significantly better in the NIR region. In the NIR region, it is also possible to separate the bands attributed to the free terminal OH or NH groups of polymeric species from those caused by their free OH or NH groups.
6. The first overtone bands of the OH and NH stretching modes of monomeric species are significantly more intense than the equivalent bands of polymeric species due to higher anharmonicity. On the other hand, due to a greater separation of the X-H charges (-δX-H+δ-:Y) in a hydrogen bond, the fundamental bands originating from the OH and NH stretching modes of polymer species are significantly more intense than those of monomeric species. While a band originating from monomeric species is significantly weaker than a large feature caused by polymeric species in the IR spectrum, the reverse is true in the NIR spectrum. Therefore, rather than in the visible spectrum, the dissociation process from polymeric to monomeric species can be more easily observed in the IR region through the first overtone of the OH or NH stretching mode of monomeric species [13].

#### 2.2.2. Benefits of NIR Spectroscopy

Firstly, NIR spectroscopy is an effective technique for non-destructive in situ examination. NIR spectroscopy allows the interior of a material to be explored. In addition, it allows contactless and fiber optic-based analysis. Thirdly, RM spectroscopy can be used on materials of different thicknesses, shapes, and states.

The advantages of NIR spectroscopy over IR spectroscopy can be discussed:

1. With a material still in its original form, NIR spectroscopy allows for in situ examination. For in situ study in IR spectroscopy, attenuated total reflection (AIR) or photoacoustic spectroscopy (PAS) can be used, but if you want to collect an absorption spectrum over the entire surface of an apple or a human head, NIR spectroscopy is the only option. It is also suitable for non-destructive processing of thick samples.
2. Because the strength of water bands is substantially weaker in the NIR spectrum than in the IR spectrum, NIR spectroscopy is generally more suitable for investigating aqueous solutions than IR spectroscopy. Aqueous solutions can be examined using AIR-IR spectroscopy, but NIR spectroscopy can investigate them in more sophisticated ways.
3. The fiber optic probe can be placed in a hazardous situation and controlled remotely. This is one of the factors that makes NIR spectroscopy suitable for online analysis. IR light fibers are substantially more expensive and less durable.
4. The length of the light path in NIR spectroscopy can be chosen extremely freely. IR spectroscopy, on the other hand, typically requires a cell with a relatively short path length. A 0.1 mm cell, a 1 cm cell, or even a 10 cm cell can be used when performing NIR spectroscopy.
5. NIR optical materials are less expensive than IR optical materials. Glass cells are a good example [13,14].

#### 2.2.3. NIR in Vein Visualization

Under certain circumstances, a molecule absorbs IR light when exposed to it. The energy difference between one vibrational energy level in a molecule (containing energy Em) and another vibrational energy level in a molecule (containing energy En) is equal to the energy hυ of the absorbed IR light (1). When expressed as an equation:

*hυ* = *E_n_* − *E_m_* (1)

This equation is called the “Bohr frequency condition.” In other words, IR light absorption occurs as a result of a change in the energy level of a molecular oscillation. As a result, the vibrational spectrum of a molecule can be found in an IR absorption spectrum [13,15].

The wavelength range between 650 and 1100 nm, commonly referred to as the “optical window” in tissues, is relatively free for NIR radiation in soft, diffusing tissues. The optical absorption and scattering properties of the skin have been used to calculate the penetration depth of photons for non-invasive blood glucose monitoring. In addition to measuring blood glucose, there are additional unique biomolecules that are quite interesting for tracking abnormalities in body homeostasis. Natural pigments have distinct absorption spectra that fall within the visible and SW-NIR spectral ranges. Examples include hemoglobin, which is a key component of red blood cells, myoglobin, which is present in muscles, and cytochromes, which are commonly found in tissues. Their spectra depend on the degree of oxygenation of the heme protein and the redox state of the cytochrome. Spectroscopy can therefore tell us in real time how oxygen is being supplied to tissues [16,17].

To demonstrate the possibilities of quantitative spectroscopy in a variety of spectral ranges, from visible to SW-NIR, the absorptions of hemoglobin in both oxygenated and deoxygenated states are presented. Continuous and non-invasive measurement methods are of particular interest. Since its initial introduction in specialized clinics for intensive care of newborns or for monitoring during labor, brain monitoring has found many applications. Studies on muscle oxygenation have also attracted considerable attention in the past [16,17].

NIR spectroscopy of the skin has been the focus of numerous studies, including those on skin transplantation, plastic and reconstructive surgery, treatment of catastrophic injuries, and wound healing. Particular emphasis is placed on regional and temporal fluctuations in skin tissue oxygenation, which can be measured continuously using NIR spectroscopy [1,3,17].

The distinctive quality of NIR radiation is that it can reach biological tissue to a depth of approximately 3 mm, where veins are located. Compared to veins, arteries are deeper, and NIR is limited to 3 mm of penetration. In addition, deoxygenated hemoglobin (Hb) in venous blood absorbs more radiation from this spectrum than surrounding tissue, unlike arterial blood, which is mainly occupied by oxygenated hemoglobin (HbO2). Hb and HbO2 can absorb NIR 1ight in the specter of 600–1300 nm. When the wavelength exceeds 600 nm, the absorption coefficients of both oxygenated and deoxygenated blood decrease significantly, but venous blood still has a higher absorption capacity in this range. In addition, HbO2 has a peak at approximately 760 nm, and the difference in absorption between the two types of hemoglobin is most noticeable between 700 and 900 nm. Therefore, it is essential that the wavelength of the NIR light source be set between 700 and 900 nm or, optimally, at 760 nm [1,3,17].

### 2.3. Medical Image Processing

#### 2.3.1. Digital Image

The practice of processing photographs on a digital computer is called digital image processing. Images play the most important role in human perception, which is not surprising, given that sight is the most developed of our senses. On the other hand, imaging devices cover almost the entire electromagnetic (EM) spectrum, from gamma rays to radio waves, unlike humans, who are limited to the visual range. They can also work with images produced by sources that humans do not usually associate with images. These consist of computer-generated images, electron microscopy, and ultrasound. Thus, a wide range of applications for digital image processing is covered [18].

#### 2.3.2. Image Processing

Image processing was developed in response to three major image-related problems: digitizing and encoding images to facilitate image transmission, printing, and storage; enhancing and restoring images for easier interpretation of images of other planets’ surfaces taken by various probes; segmenting and describing images, as a fundamental step in the field of machine vision. Currently, digital image processing is the main focus of image processing [19].

#### 2.3.3. Image Processing in Medicine

Medical image processing is the process of creating specialized methods for improving raw medical image data for further analysis and specific visualization. Applied mathematics, computer science, engineering, statistics, physics, biology, and medical experts have contributed to the rapid growth and interdisciplinary nature of the field of research. Computerized diagnosis is already a significant component of daily clinical practice. The use of different imaging modalities and the avalanche of new technological advances raise several issues, such as how to process and analyze a considerable number of images to create high-quality information for the diagnosis and treatment of diseases [18].

#### 2.3.4. Adaptive Histogram Equalization

This technique is only executable if the image is converted to grayscale. “The histogram is the graphical representation of the number of pixels for each intensity value.” Sometimes, the histogram of local regions of an image can be heavily skewed toward one end of the grayscale, while the histogram of the entire image shows a wide distribution. Although, under these conditions, it is often desirable to increase the contrast of these local areas, global histogram equalization is useless. By using adaptive histogram equalization, this can be achieved. The term “adaptive” means that, depending on local properties, different portions of the image are processed differently (i.e., different lookup tables are used) [20].

Adaptive histogram equalization comes in a variety of forms, but the so-called sliding window (or tile) approach is probably the simplest and most widely used. This approach divides the base image into relatively small adjacent “tiles” or local N × M neighborhood sections (e.g., 16 × 16 pixels). The lookup table for histogram equalization is calculated for each inner window using the larger outer window surrounding each inner window [20].

In general, this works well to enhance local contrast, but separate processing of each of these regions can lead to the appearance of “block artifacts,” and artifacts at the boundaries between interior windows can give the impression that the image is composed of several blocks that are only slightly misaligned. In most cases, artifacts can be reduced by enlarging the outer window relative to the inner window [20].

A different approach to adaptive histogram equalization has been used effectively and is attributed to Pizar. The steps are as follows:

1. A regular grid of points is superimposed on the image. In this method, the spacing of the points in the grid varies, but is often a few tens of pixels.
2. For each point of the grid, a rectangular window with a double distance from the grid is calculated. Thus, a given window has a 50% overlap with its immediate neighbors to the north, south, east, and west.
3. For each of these windows, a lookup table with the equalized histogram is calculated. Each pixel in the image is included in four neighboring rectangular windows, or four neighborhoods, due to the 50% overlap of the windows.
4. The converted value of a given image pixel is determined by combining the weighted output values from the four neighborhood lookup tables, according to the bilinear formula (2) shown below:

*I* = (1 − *a*)(1 − *b*)*I*_1_ + *a*(1 − *b*)*I*_2_ + (1 − *a*)*bI*_3_ + *abI*_4_ (2)

It should be noted that this formula gives appropriate weight to the areas to which calculation point I belongs most. For example, a pixel located exactly on a grid point obtains its equalized value only from its surrounding neighborhood, while a point that is equidistant from the four nearest grid points is an equally weighted combination of all four surrounding neighborhood values. The fact that neighborhoods overlap is something that must be considered.

A final extension of this method is the adaptive equalization approach to limited contrast histograms [19,20].

#### 2.3.5. Adaptive Equalization of Contrast-Limited Histogram

In general, histogram calculations of local regions that equalize intensity values within certain regions tend to increase contrast too much and accentuate noise. The intensity histogram of certain areas of an image can produce unfavorable results because these areas are naturally smooth and have low actual contrast [18].

Contrast limiting is mainly used to set a ceiling on the total (normalized) contrast increase that applies at any given location [20].

The local contrast of the image can sometimes be significantly improved by adaptive histogram equalization. However, there is no general theory to help us, because each image is fundamentally distinct from others, the number of plates into which the image is divided can be changed, and the probability distribution of the target has a specific shape. Thus, to a large extent, obtaining an image with adequate contrast characteristics is an art in which these parameters are modified experimentally [20].

#### 2.3.6. Median Filter

The median filter is another commonly used filter. The median filtering overcomes several limitations of the mean filter, albeit at a higher computational cost. Instead of the mean, each pixel is approached by the statistical median of its N x M neighborhood. Clear high-frequency details (edges) are better preserved by the median filter than by the mean filter, and noise is also reduced, especially isolated fog spikes (also known as “salt and pepper” noise) [20].

The middle of the ordered distribution of values, the median m of a set of numbers is the number for which half the numbers are less than it and half are greater. The median is more resistant to outliers and does not produce a new irrational pixel value, as it is a pixel value derived from the neighborhood of the pixel itself. This helps prevent loss of image detail and blurring of edges [20].

By definition, the median operator requires the values in the neighborhood of the pixel to be sorted at each individual pixel location. For this reason, the median operator now requires more calculations [20].

## 3. Materials and Methods

### 3.1. Code

Below, we will explain step by step how the programming for the vein detection device was carried out using MATLAB (MathWorks, Natick, MA, USA), version R2022b.

#### 3.1.1. Image Acquisition

In the first phase of the project, the aim was to create a connection between the Raspberry Pi and the computer, using the interface provided by MathWorks, MATLAB. However, during testing, it was discovered that MATLAB has connectivity errors with Raspberry Pi, which are currently being resolved. These errors include the inability to install the program created by the MathWorks team for the Raspberry Pi model on a micro-SD card, even though different micro-SD cards were tried, and even updating the program and libraries. This was confirmed by the fact that the new version of MATLAB, MATLAB 2023a, which was also installed to resolve the previous error, no longer offers the option to install the Raspbian Image interface for boards. The second error encountered comes from the Raspberry Pi team, who did not design their current interface to be compatible with MATLAB so that the program commands do not correspond to the board’s directories and files, even after reinstalling the directories and updating the libraries. For these reasons, it was necessary to create a code for the device’s microcontroller, in this case Raspberry Pi 4 Model B. This code will allow us to acquire photos directly from the Raspberry Pi board interface. The package already installed on the board acts like a computer, on which other programs can be installed. To be able to work from the board, Python 3.9.2 was also installed on it, in which the necessary code for the camera was created.

A second step in the connection was to connect the Raspberry Pi Camera Module 2 NoIR to the Raspberry Pi board interface and Python, where the necessary configurations were made to allow the camera to be started from the program. After that, a specific code was used to determine all the camera’s configuration and available input information and then to start the camera, set its parameters, and capture images with it. Then with the help of a portable USB flash drive the obtained images were transferred to the computer containing the MATLAB program for the next phase. The current implementation should be considered an offline prototype. Images were acquired using the Raspberry Pi camera system and then processed in MATLAB on a separate computer. Therefore, the present study demonstrates the feasibility of the acquisition and processing workflow, but not real-time operation. Future development will focus on transferring the processing pipeline to an embedded or open-source implementation suitable for real-time visualization. MATLAB was used in this study as a prototyping and analysis environment. For a final low-cost implementation, the same processing steps could be transferred to an open-source platform, such as Python with OpenCV, or implemented directly on embedded hardware.

#### 3.1.2. Image Preprocessing

**Figure 2:**
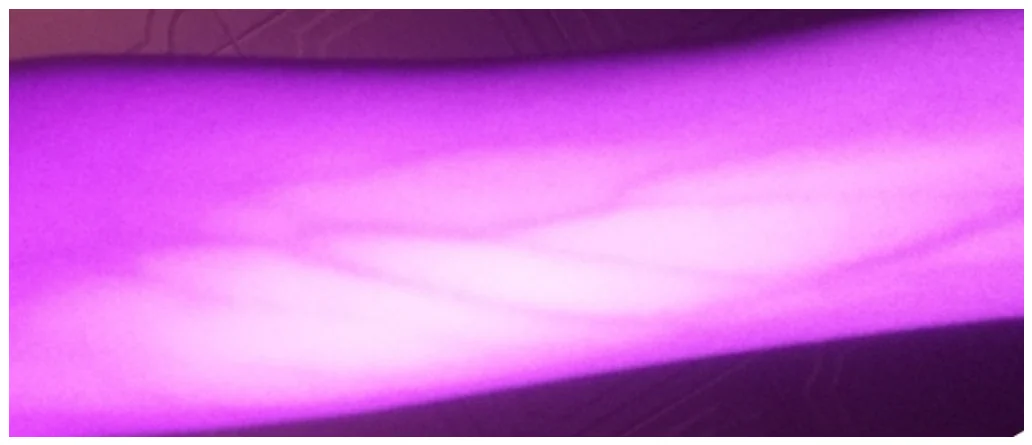
Representative original near-infrared (NIR) image of the forearm acquired from a study participant and used as the input image for the development and application of the digital image-enhancement filters.

To create filters for image enhancement, the program captured an image of the forearm of one of the subjects on whom the physical device was tested, using a camera equipped with NIR technology. This image was named “Original Image” (Figure 2), and all the digital filters created were applied to it.

**Figure 3:**
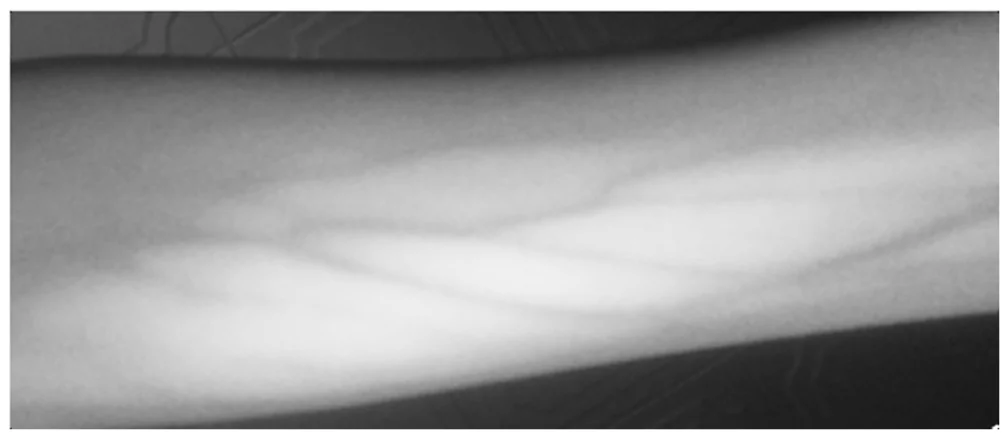
Forearm image after grayscale conversion and the first median-filtering step using a 3 × 3 neighborhood. Median filtering was applied to reduce image artifacts and noise prior to subsequent image-enhancement processing.

In the case of image preprocessing, the first thing that was done was to convert the image from color to grayscale, check the number of channels it had, and modify it where necessary to only one channel, followed by the first median filtering, which was performed using the default 3 × 3 neighborhood to reduce the artifacts already present in the image, which appeared for various reasons, Figure 3.

**Figure 4:**
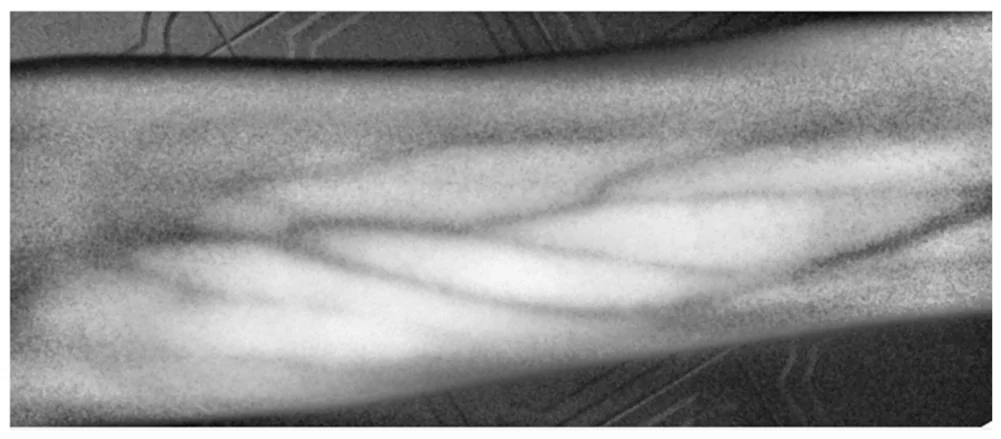
Forearm image after application of adaptive histogram equalization. This processing step was used to enhance local contrast by redistributing grayscale intensity values within different regions of the image.

Next, adaptive histogram equalization was applied to the new image to check each area of the image and modify the distributions in the gray range of the histogram of that area to increase the contrast in any section of the image, Figure 4. When adaptive histogram equalization was used, MATLAB’s default CLAHE settings were applied, corresponding to an 8 × 8 tile grid, a uniform distribution, 256 bins, and a clip limit of 0.01.

**Figure 5:**
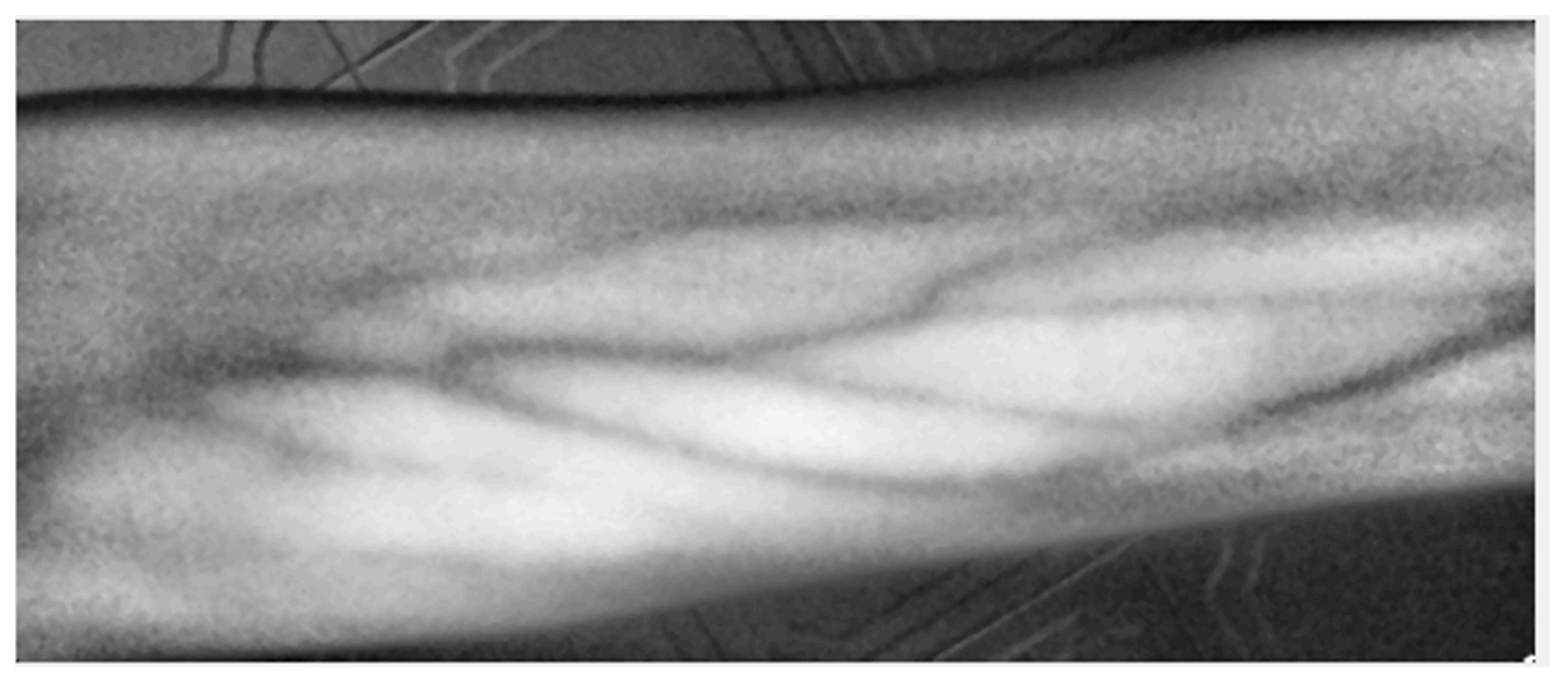
Forearm image after the second median-filtering step. This filtering step was applied to reduce artifacts introduced during adaptive histogram equalization while preserving the enhanced image contrast.

As can be seen in the illustration above, although the contrast level was modified correctly, making the image much clearer, an adverse effect of equalization is the appearance of new artifacts in the image. To correct them, a second median filtering was necessary, Figure 5.

#### 3.1.3. Vein Extraction

**Figure 6:**
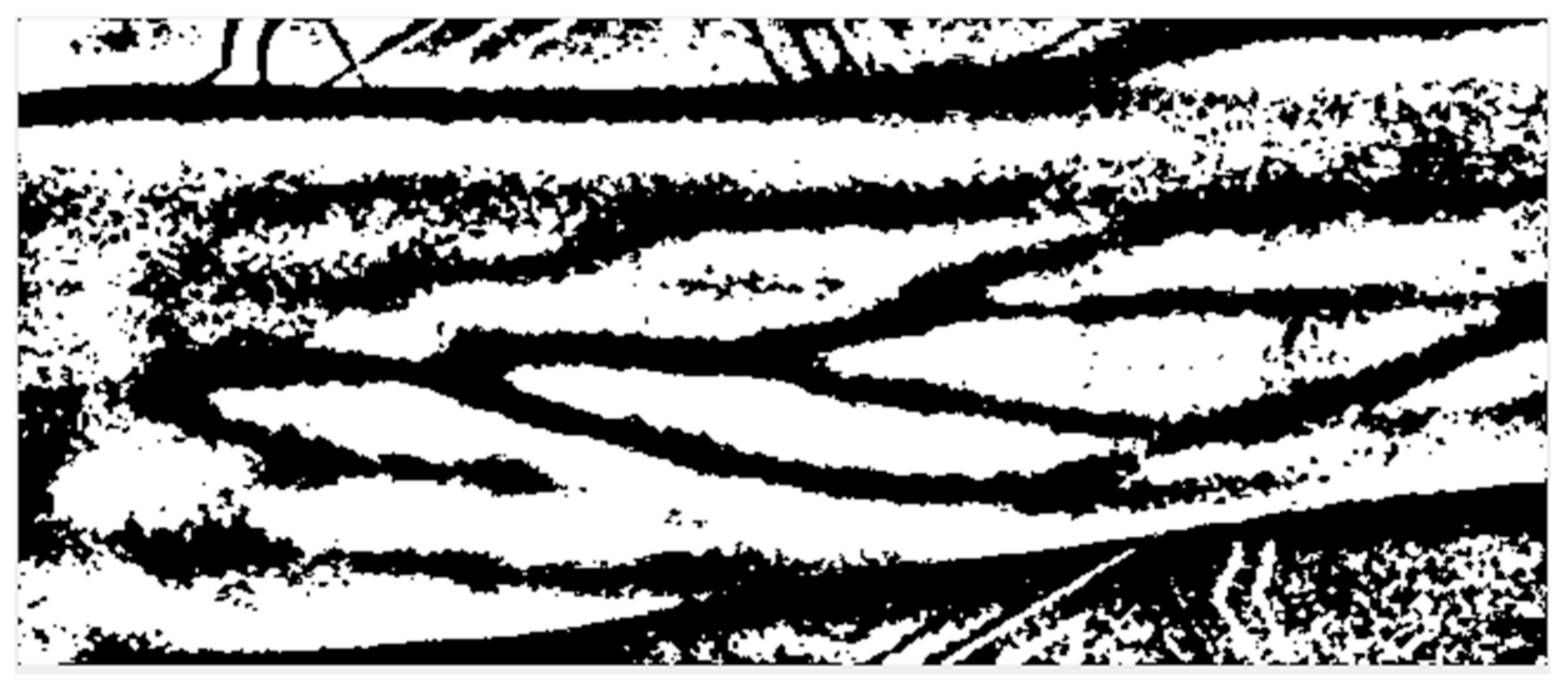
Binarized forearm image obtained using adaptive thresholding. The processed image was converted to a binary black-and-white representation using a sensitivity value of 0.6, bright foreground polarity, and the local mean statistic to improve the delineation of superficial veins.

To extract the veins easily, the image with the second filtering (Figure 6) was binarized, in non-colors only black and white, using an adaptive threshold that was applied to each pixel of the image, corresponding to a sensitivity value of 0.6, a neighborhood size calculated as $2*\mathrm{floor}\left(\frac{\mathrm{size}\left(I\right)}{16}\right)+1$, bright foreground polarity, and the local mean statistic, the veins being much better outlined by this method, as can be seen in Figure 6.

**Figure 7:**
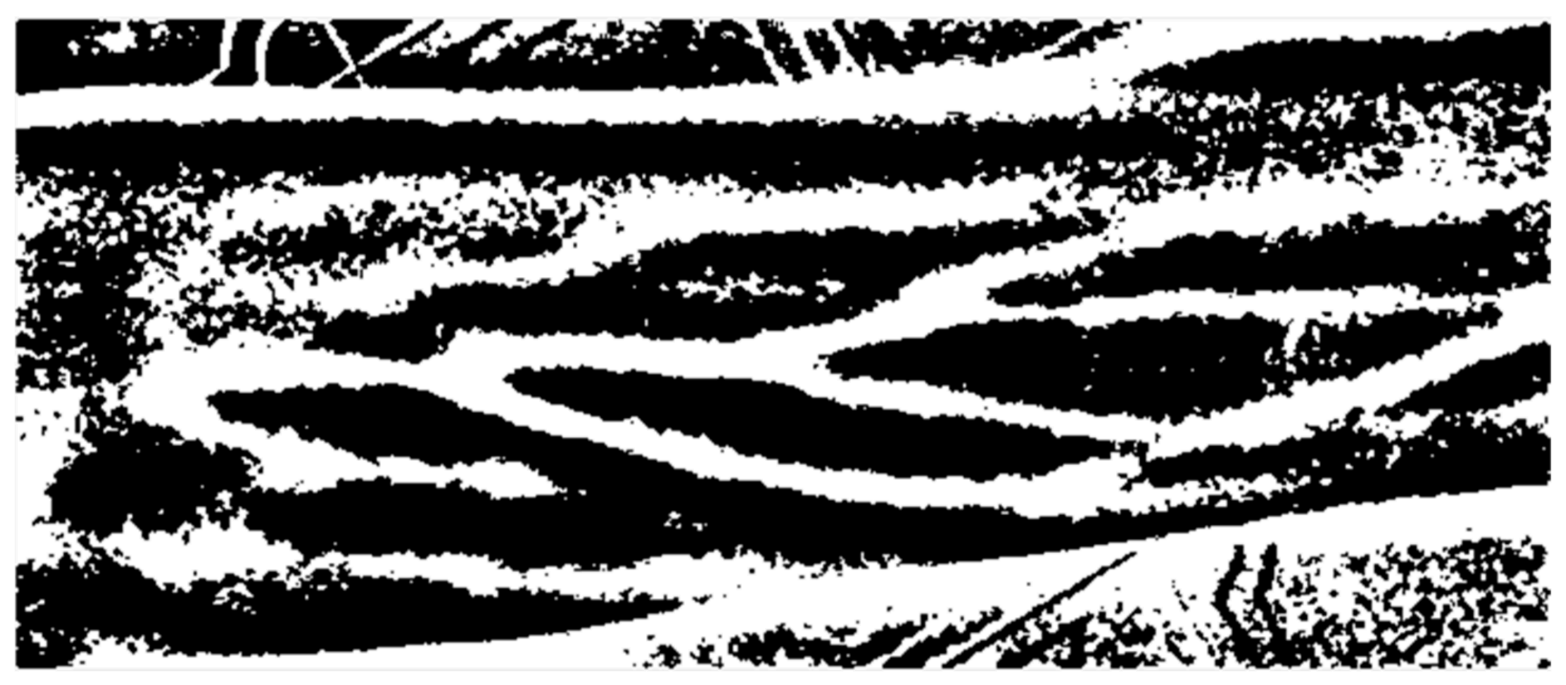
Inverted binary forearm image obtained by reversing the binary pixel values of the thresholded image. This inversion changed the vein regions from black to white and the surrounding background from white to black, facilitating subsequent image-processing steps.

From the image above, during binarization, the veins took on the non-color black, and the rest of the surfaces took on the color white. This is not good for our filter, so the decision was made to invert the image by reversing the binary pixel values so that 0 becomes 1 and 1 becomes 0, Figure 7.

**Figure 8:**
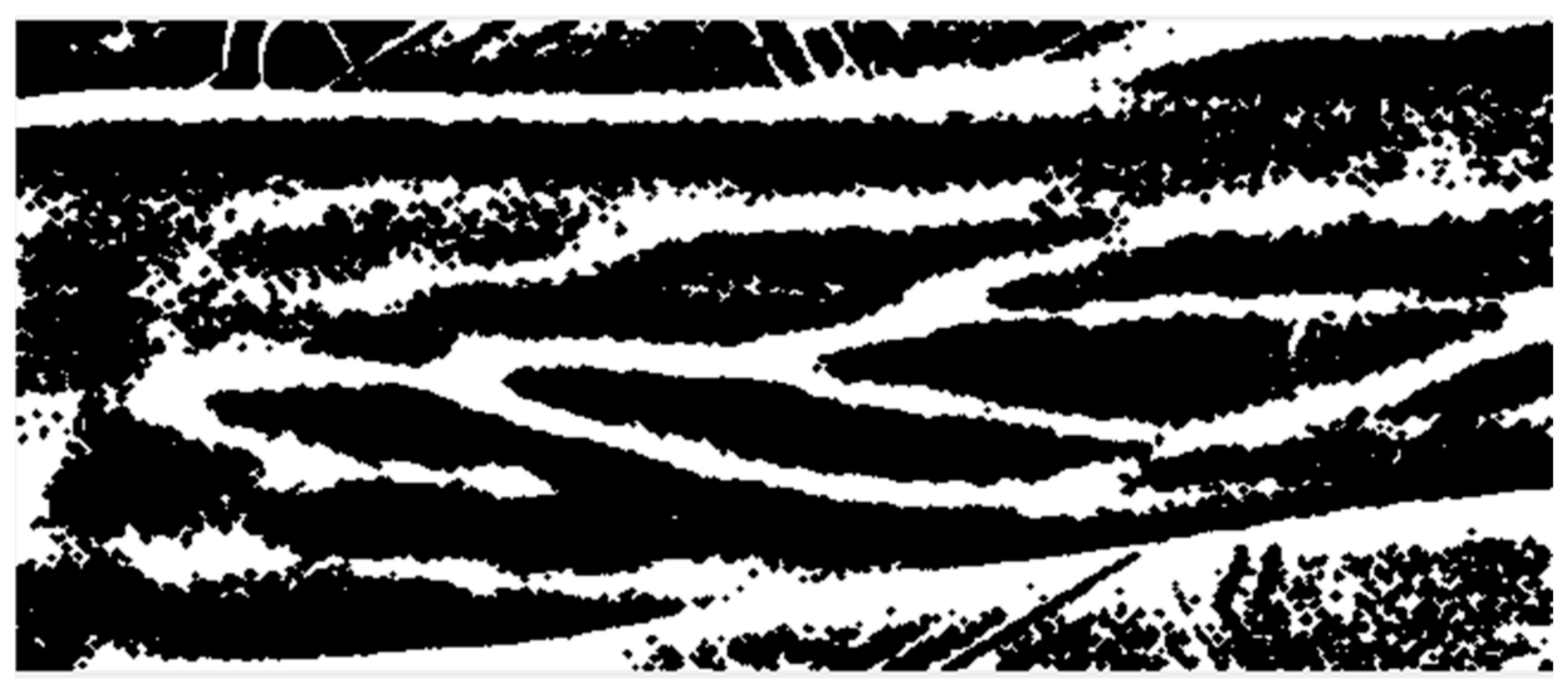
Forearm image after morphological erosion using a disc-shaped structuring element with a radius of 1 pixel. This operation reduced the thickness of the segmented vein regions to improve their delineation for subsequent image-processing steps.

For now, the veins can be easily seen in the new image, but to achieve greater accuracy, morphological erosion was applied to it using a disc-shaped structured element with a radius of 1, resulting in an overall thinning of the veins, Figure 8.

**Figure 9:**
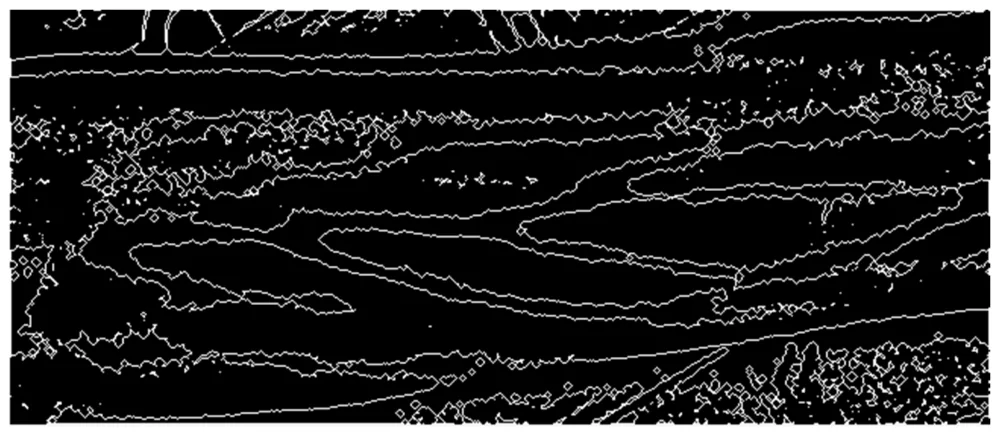
Perimeter detection of the segmented vein regions using MATLAB’s default 4-connectivity for two-dimensional images. The detected boundaries were used to visually assess the continuity and regularity of the segmented venous structures.

Considering all the changes that the image has undergone, for a final check, the perimeter of the white areas, i.e., the veins, with MATLAB’s default 4-connectivity for 2-D images, was determined to verify whether there was any irregularity, as illustrated in Figure 9.

**Figure 10:**
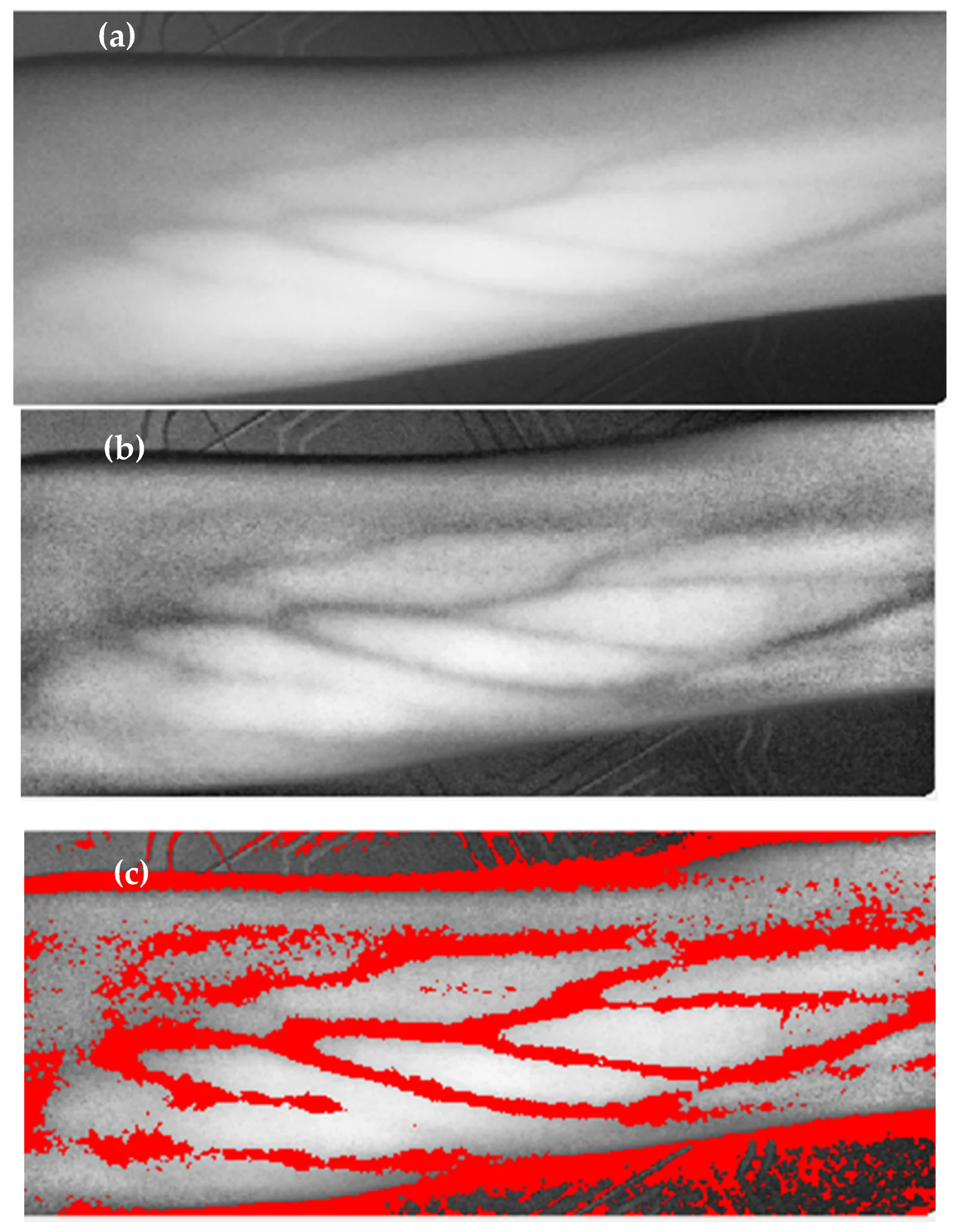
Comparison of the forearm image at different processing stages: (**a**) original grayscale image; (**b**) image after adaptive histogram equalization; and (**c**) equalized image with the segmented vein mask overlaid in red. The overlay was generated using the eroded binary image as the vein mask to visualize the effectiveness of the image-processing pipeline.

In order to observe the effectiveness of all the functions applied to the image, an overlay was applied to the equalized image, using the eroded image as a reference. A comparison between the original image, the histogram-equalized image, and the image with the mask created can be found in Figure 10.

The selected processing parameters were used to establish a reproducible baseline workflow for the prototype rather than a fully optimized segmentation algorithm. MATLAB default settings were retained for several steps to improve reproducibility and avoid excessive manual tuning on a small dataset. The adaptive threshold sensitivity and erosion radius were selected during preliminary testing to preserve visible vein structures while reducing small background artifacts. A systematic parameter optimization study was outside the scope of the present technical feasibility evaluation and will be included in future work. The present study did not include a full ablation comparison between alternative processing pipelines. The selected sequence was used as an initial reproducible workflow for prototype testing. Future work will compare the full pipeline with CLAHE alone, median filtering plus CLAHE, alternative thresholding methods, vessel-enhancement filtering, and learning-based segmentation approaches.

For contrast and CNR calculation, the anatomical zone to be analyzed was first selected in the MATLAB code, corresponding to the examined region for each participant. Within the selected image, the region of interest corresponding to the vein area was then manually selected by the operator on the raw near-infrared image. One ROI was placed over a visible superficial vein segment, and one adjacent ROI was selected from nearby background tissue without visible vascular structures. The background ROI was chosen close to the vein ROI to reduce the influence of non-uniform illumination. The same selected regions were then used for the corresponding processed image, allowing a direct before-and-after comparison. ROI dimensions were kept similar across image pairs where possible. Because the regions of interest were selected manually by the operator, ROI selection may introduce observer-dependent variability. This aspect was minimized by applying the same selected regions to the corresponding unprocessed and processed image pair, allowing a direct comparison under identical ROI conditions.

### 3.2. Device

**Figure 11:**
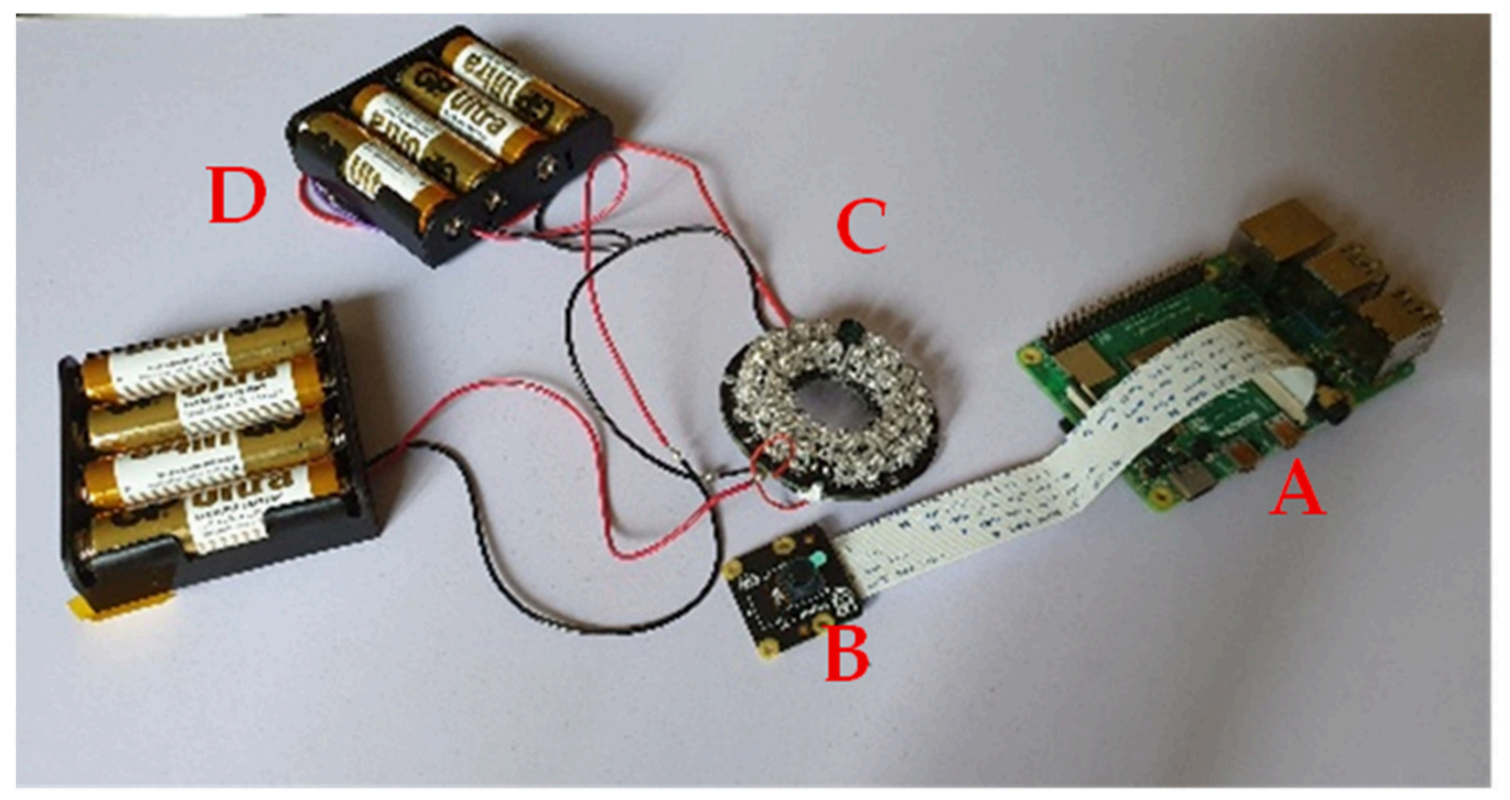
Main hardware components of the proposed near-infrared vein-detection device: (A) Raspberry Pi 4 Model B single-board computer; (B) Raspberry Pi Camera Module 2 NoIR; (C) 36-LED, 960 nm infrared illumination module; and (D) battery holders used to provide the 12 V power supply required by the infrared LED module.

A primary component of the physical device, which serves as the “brain” of the newly created apparatus, is the single-board computer, the “Raspberry Pi 4 Model B” board, with 4 GB of RAM, Figure 11A.

A micro-SD card will be used to load the program necessary for the board to function, and a 4 GB portable USB memory stick will be used to transfer the acquired photos from the Raspberry Pi to the computer so that they can be loaded into the code created using the MATLAB interface on the board. The decision was made to use this microcontroller because it is capable of processing images captured at a higher resolution and is compatible with a camera that does not have an integrated infrared light filter, a very important specification for this project.

A second component is the camera provided by Raspberry Pi, Camera Module 2 NoIR, Figure 11B. This was used because it allows the capture of images in the infrared wave spectrum, as it is designed without the infrared light filter that exists in almost all other cameras. In terms of specifications, the camera has an 8-megapixel image sensor created by Sony, which can support videos with a quality of 1080p30, 720p60, and 640 × 480p90. It was connected through its dedicated port in the Raspberry Pi.

The third component of the physical project is the 36-LED 960 nm 12 V infrared module, which will illuminate the examined anatomical region so that the distribution of veins can be visualized, Figure 11C. The prototype used a 960 nm infrared LED module because this wavelength was available in a low-cost LED array compatible with the proposed hardware configuration. Although wavelengths in the 700–900 nm range are commonly reported as favorable for hemoglobin-related optical contrast, the aim of this initial prototype was to evaluate whether an accessible 960 nm source could still enhance superficial vein visibility when combined with image processing. The choice of 960 nm may influence the balance between hemoglobin absorption, tissue scattering, penetration depth, and background tissue response. Therefore, future versions of the device should compare multiple illumination wavelengths, especially within the 700–900 nm range, to determine the optimal wavelength for vessel-to-background contrast.

The fourth component was a necessity for the LED module, which requires a 12 V power supply, and the Raspberry Pi can provide a maximum of 5 V, an external power supply was used. In this case, two battery holders containing 4 R6 batteries each were used, Figure 11D, with a total of 8 R6/AA 1.5 V batteries. To obtain a total of 12 V, the batteries were connected in series, with a black wire from one case connected to a red wire from the other case, and the remaining wires connected to a two-pin connector, which was then inserted into the LED module.

The physical device, consisting of the parts listed above, can be seen in Figure 11.

## 4. Results

The experimental technical evaluation of the proposed system was performed on three participants. Age, sex, and skin phototype were recorded as descriptive characteristics only and were not used for subgroup analysis, since the number of participants was not sufficient to evaluate demographic effects. The purpose of including these characteristics was to document the tested sample, not to assess the influence of age or sex on image quality. The relevant characteristics for each participant are reported individually as follows: Participant 1: age 24 years, sex male, in the Fitzpatrick classification phototype, skin type II, fair skin, BMI 20.6, anatomical region examined Anterior region of forearm, Cubital fossa, Dorsal region of hand; Participant 2: age 48 years, sex female, in the Fitzpatrick classification phototype, skin type II, light skin, BMI 23.8, anatomical region examined Anterior region of forearm, Cubital fossa, Dorsal region of hand; Participant 3: age 49 years, sex male, in the Fitzpatrick classification phototype, skin type II, fair skin, BMI 24.7, anatomical region examined Anterior region of forearm, Cubital fossa, Dorsal region of hand. This initial set of participants were chosen for the purpose of seeing what kind of changes can appear through time, participant 1 and participant 3 are the same sex but 25 years apart. And for participant 2, the role was to check if there are differences in the vein mapping between people of opposite sex but same age group. The experimental sample was limited to three healthy participants, all classified as Fitzpatrick skin phototype II. Therefore, the results should be interpreted within this restricted acquisition context. The findings describe the behavior of the prototype and processing pipeline in the tested images and should not be generalized to other skin phototypes, age groups, BMI categories, vascular depths, vascular diseases, or clinically difficult venous access populations. Testing in individuals with poor superficial vein visibility, difficult venous access, darker skin phototypes, higher BMI, advanced age, or vascular pathology represents a necessary next stage of validation.

**Figure 12:**
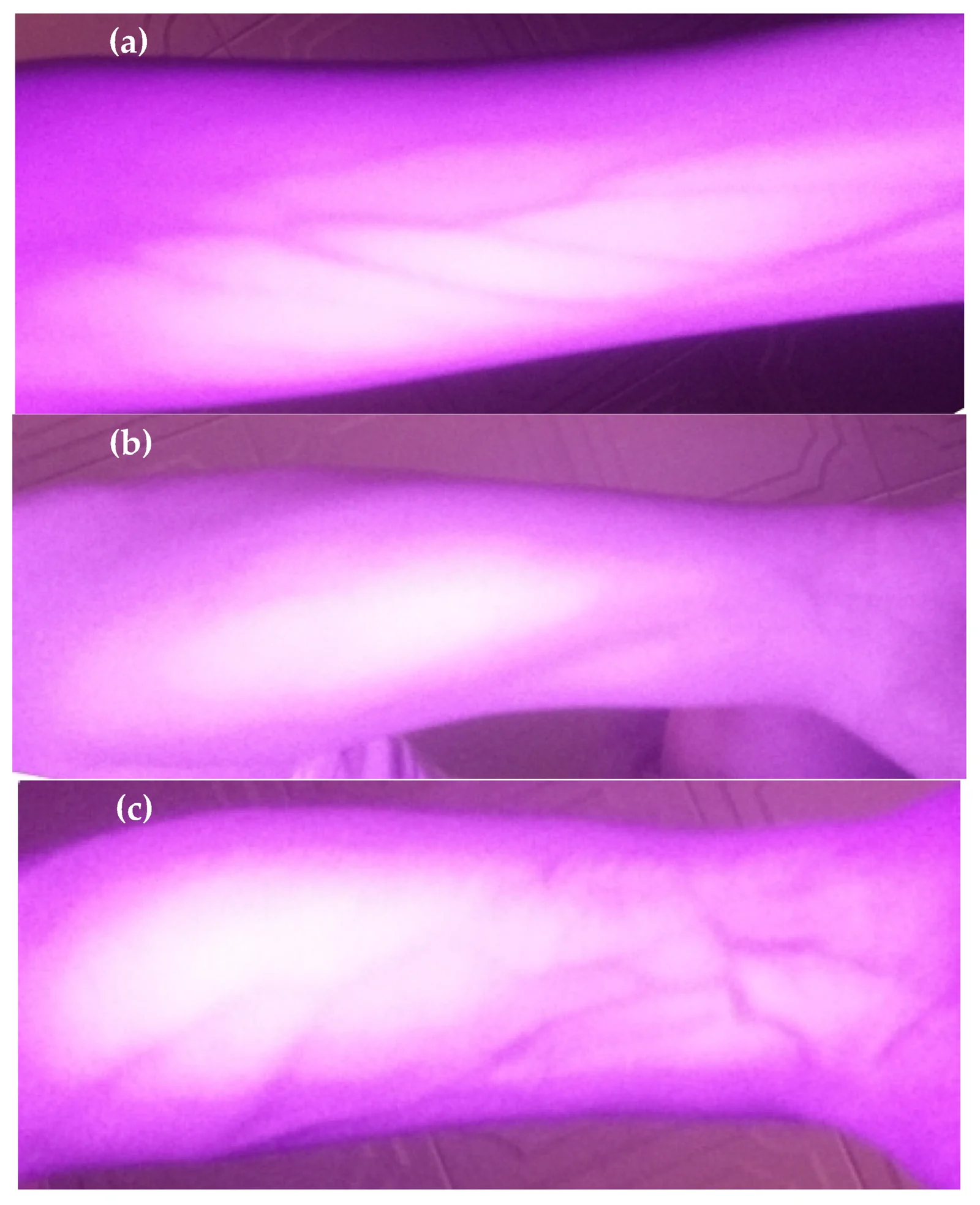
Near-infrared images of the ventral aspect of the forearm acquired using the developed vein-detection device: (**a**) Male subject, 24 years old; (**b**) Female subject, 48 years old; and (**c**) Male subject, 49 years old.

**Figure 13:**
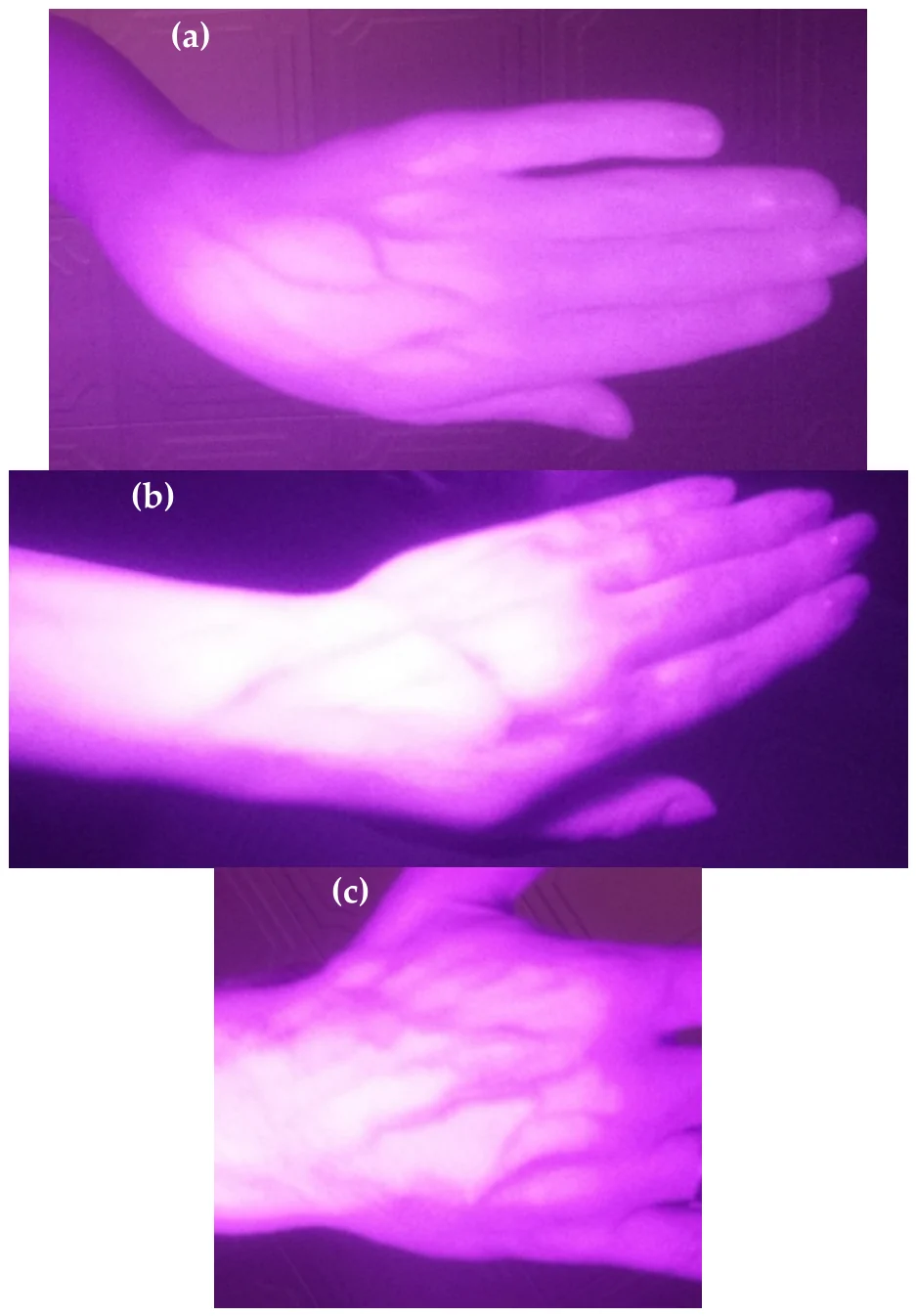
Near-infrared images of the dorsal aspect of the hand acquired using the developed vein-detection device: (**a**) Male subject, 24 years old; (**b**) Female subject, 48 years old; and (**c**) Male subject, 49 years old.

**Figure 14:**
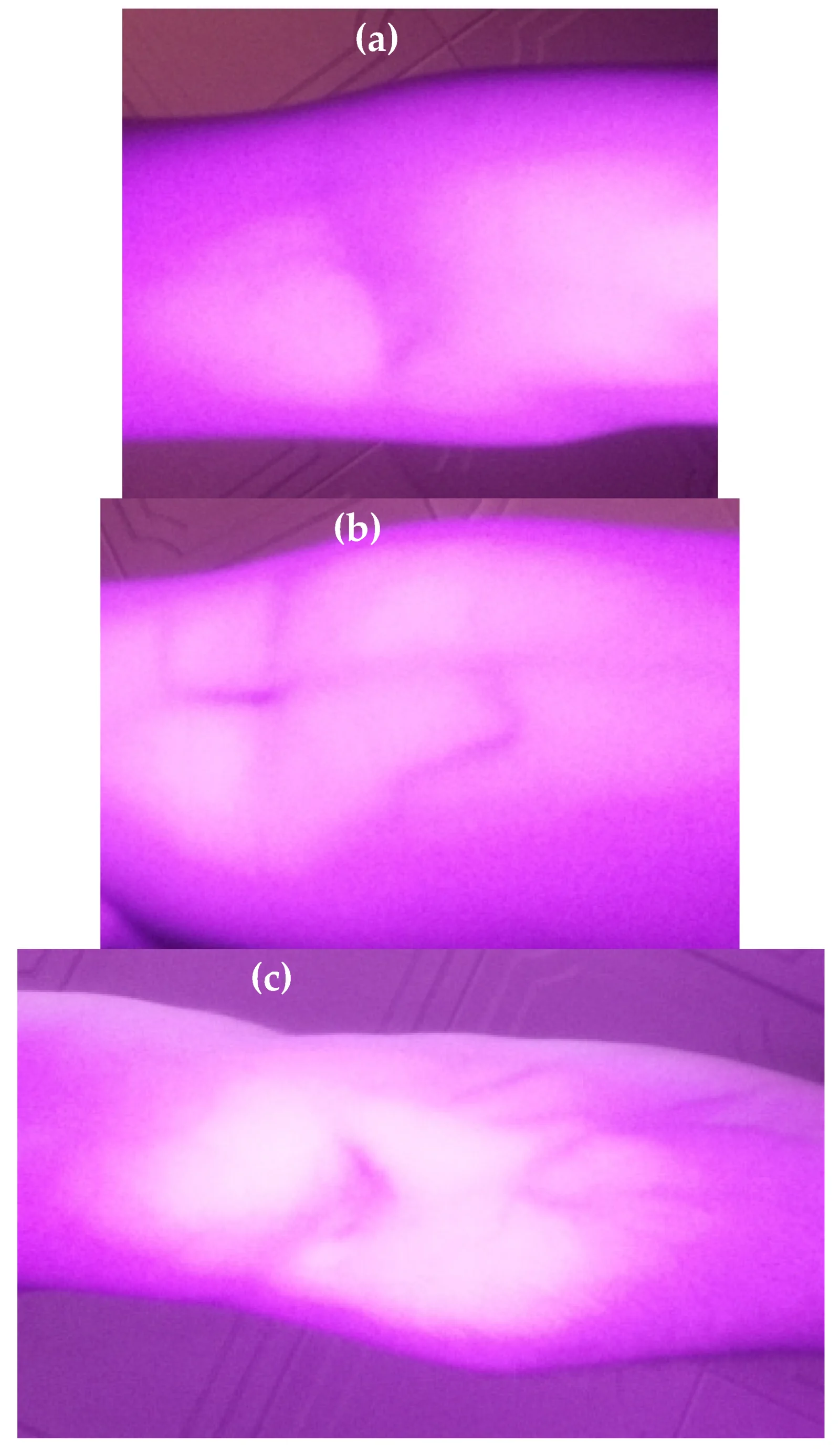
Near-infrared images of the antecubital fossa acquired using the developed vein-detection device: (**a**) Male subject, 24 years old; (**b**) Female subject, 48 years old; and (**c**) Male subject, 49 years old.

To highlight the effectiveness of the project in visualizing veins, the physical device, and the image filtering program, a set of nine images was selected from the total number of tests taken. This set of images is divided into three areas analyzed on each of the above-mentioned volunteer subjects. These areas were chosen to compare the results obtained for the palmar/ventral portion of the forearm, Figure 12, the dorsal area of the hand, Figure 13, and the antecubital fossa, or the inner part of the elbow joint, Figure 14. The entire set obtained using the camera without an infrared filter can be seen below. The antecubital fossa, dorsal hand, and ventral forearm were selected because they are superficial upper-limb regions that are accessible for image acquisition and relevant to peripheral vein visualization. These regions also allowed preliminary testing of the processing pipeline on areas with different local anatomy, surface curvature, and vein visibility. The present results should not be assumed to apply directly to other venipuncture sites without additional testing, since local tissue thickness, vein depth, skin curvature, and illumination geometry may influence image quality.

All images were acquired using the same prototype configuration under controlled indoor lighting conditions. The camera was positioned approximately 30 cm from the skin surface, with its optical axis oriented as perpendicular as possible to the examined anatomical region. The infrared LED module was positioned adjacent to the camera at approximately the same distance from the skin surface and oriented at an angle of approximately 45° relative to the examined region. The same illumination configuration was maintained for all acquisitions. Participants remained seated during image acquisition, and the examined limb was kept stable to minimize motion artifacts. All images were acquired by the same operator to reduce procedural. The irradiance at the skin surface was not measured instrumentally in this development study.

This set of images was then imported into MATLAB for preprocessing and visualizing the veins in each area of the three subjects, resulting in two additional images for each image, one in which the contrast of the images already converted to grayscale was modified using the histogram equalization function, thus highlighting the veins, while the second image contained the equalized images to which a mask was applied in order to easily map the veins.

**Figure 15:**
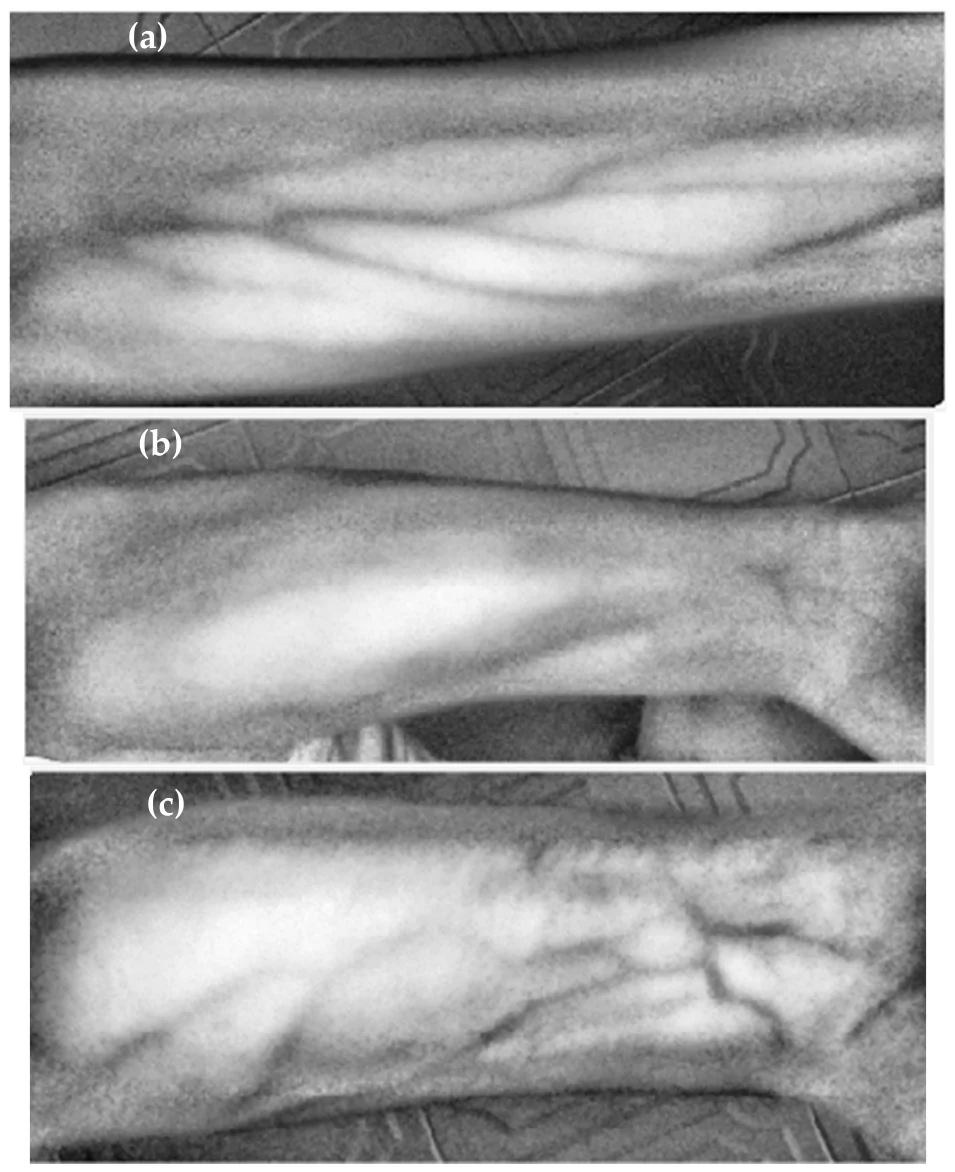
Near-infrared images of the ventral forearm after adaptive histogram equalization: (**a**) Male subject, 24 years old; (**b**) Female subject, 48 years old; and (**c**) Male subject, 49 years old.

**Figure 16:**
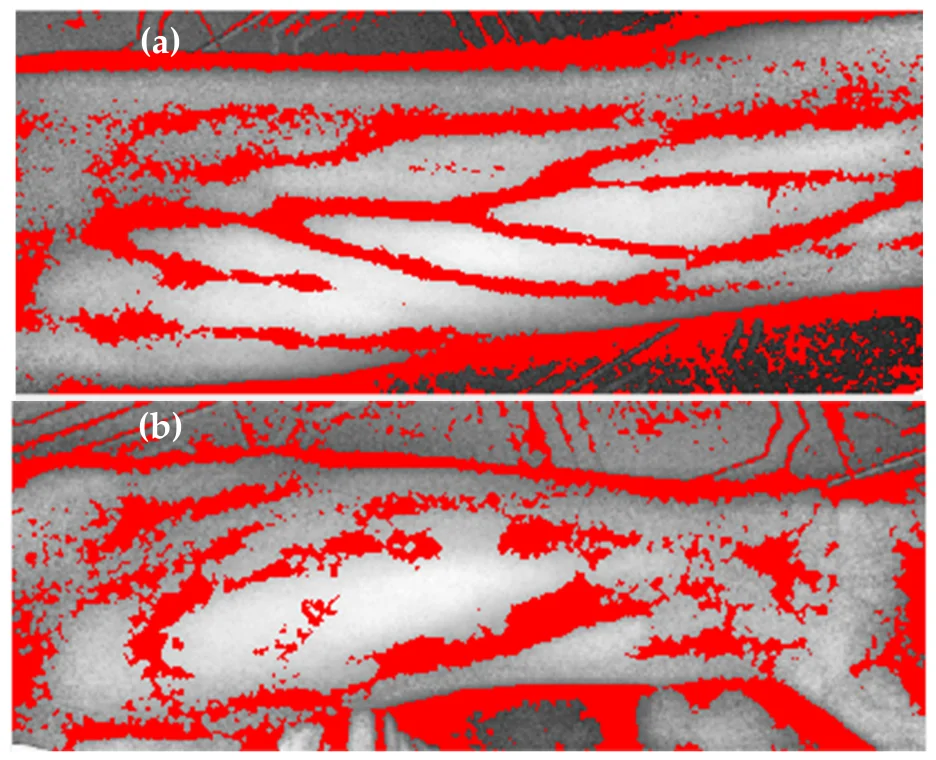

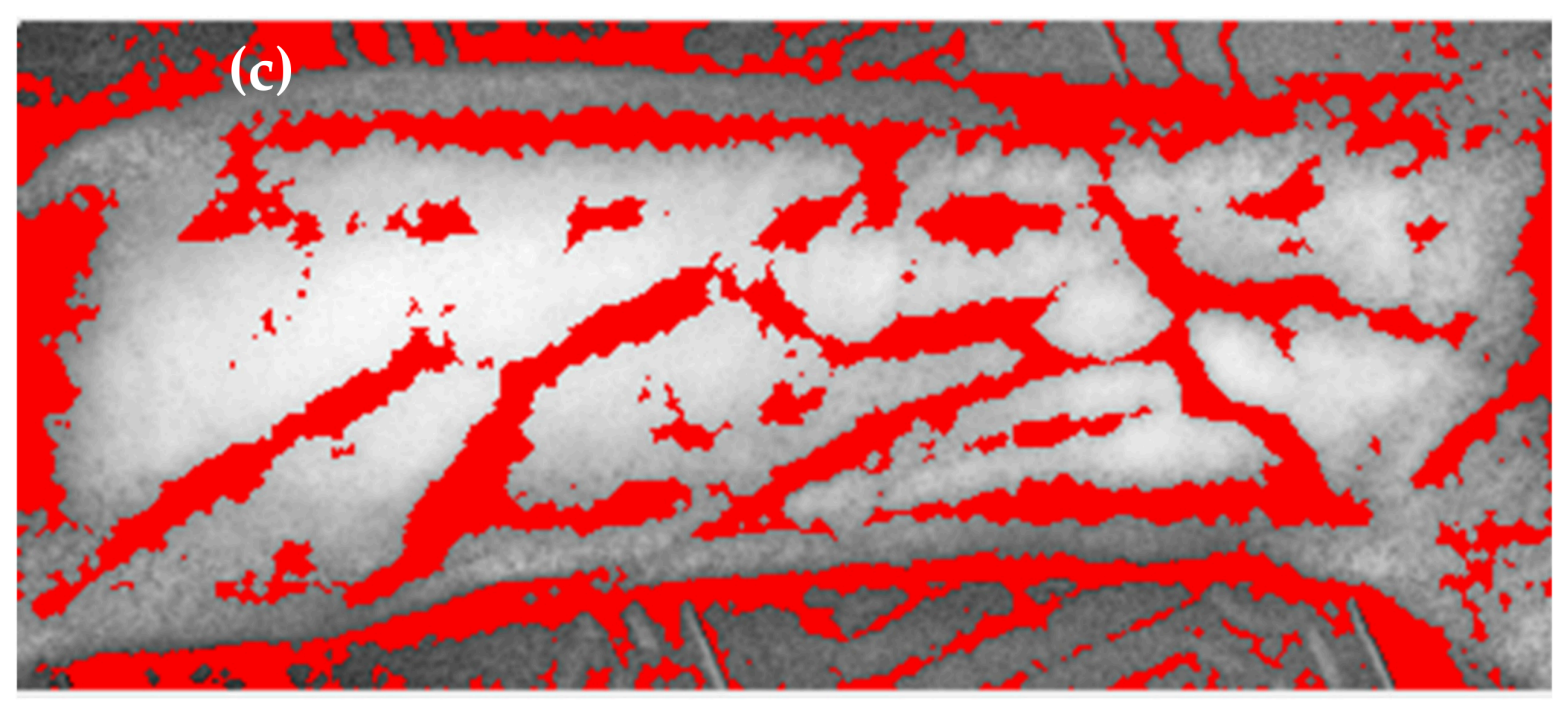
Processed near-infrared images of the ventral forearm with the segmented vein mask overlaid in red: (**a**) Male subject, 24 years old; (**b**) Female subject, 48 years old; and (**c**) Male subject, 49 years old.

For the ventral area of the forearm, after applying digital filters, histogram equalization, Figure 15, and the mask for contouring the veins, Figure 16, it can be seen that subject a, a 24-year-old male, has the most clearly defined median forearm veins, and the cephalic vein can also be seen.

**Figure 17:**
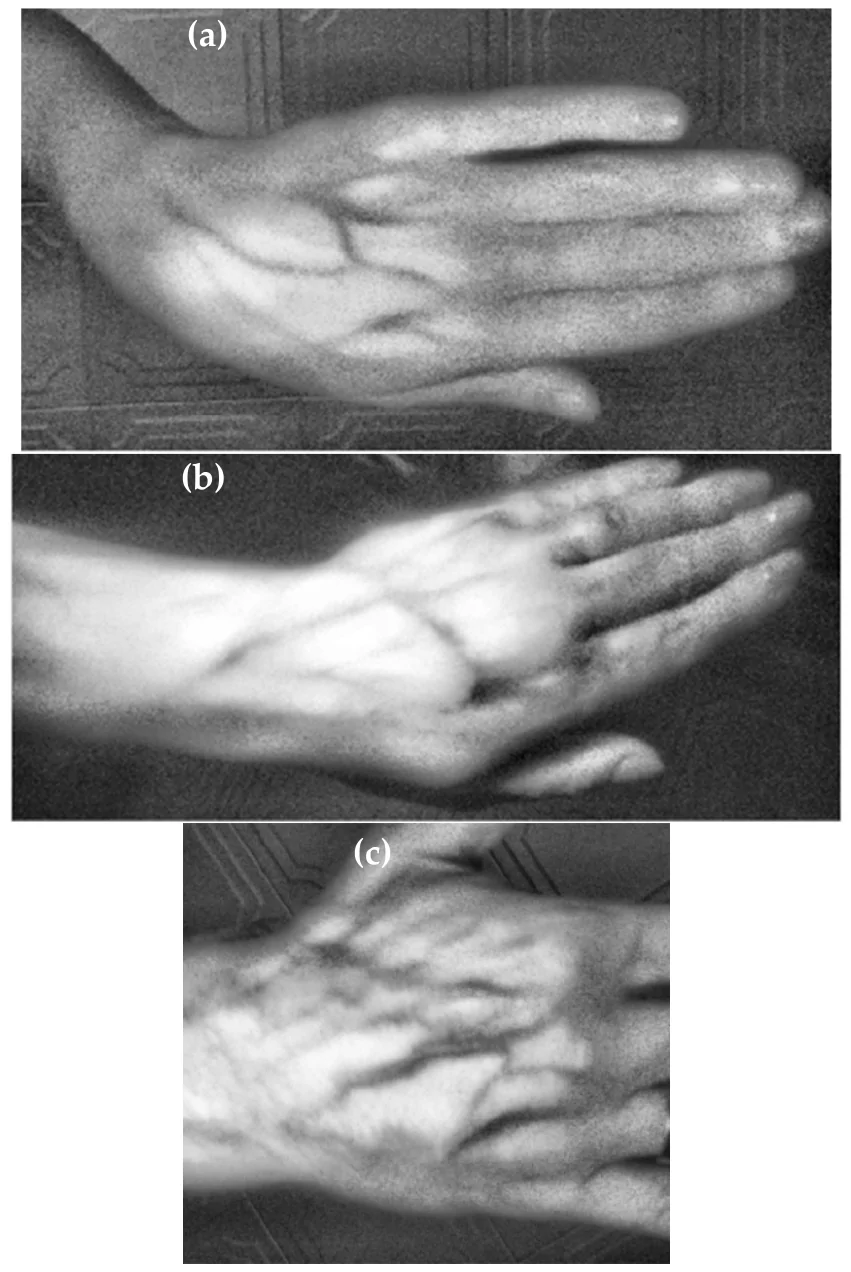
Near-infrared images of the dorsal aspect of the hand after adaptive histogram equalization: (**a**) Male subject, 24 years old; (**b**) Female subject, 48 years old; and (**c**) Male subject, 49 years old.

**Figure 18:**
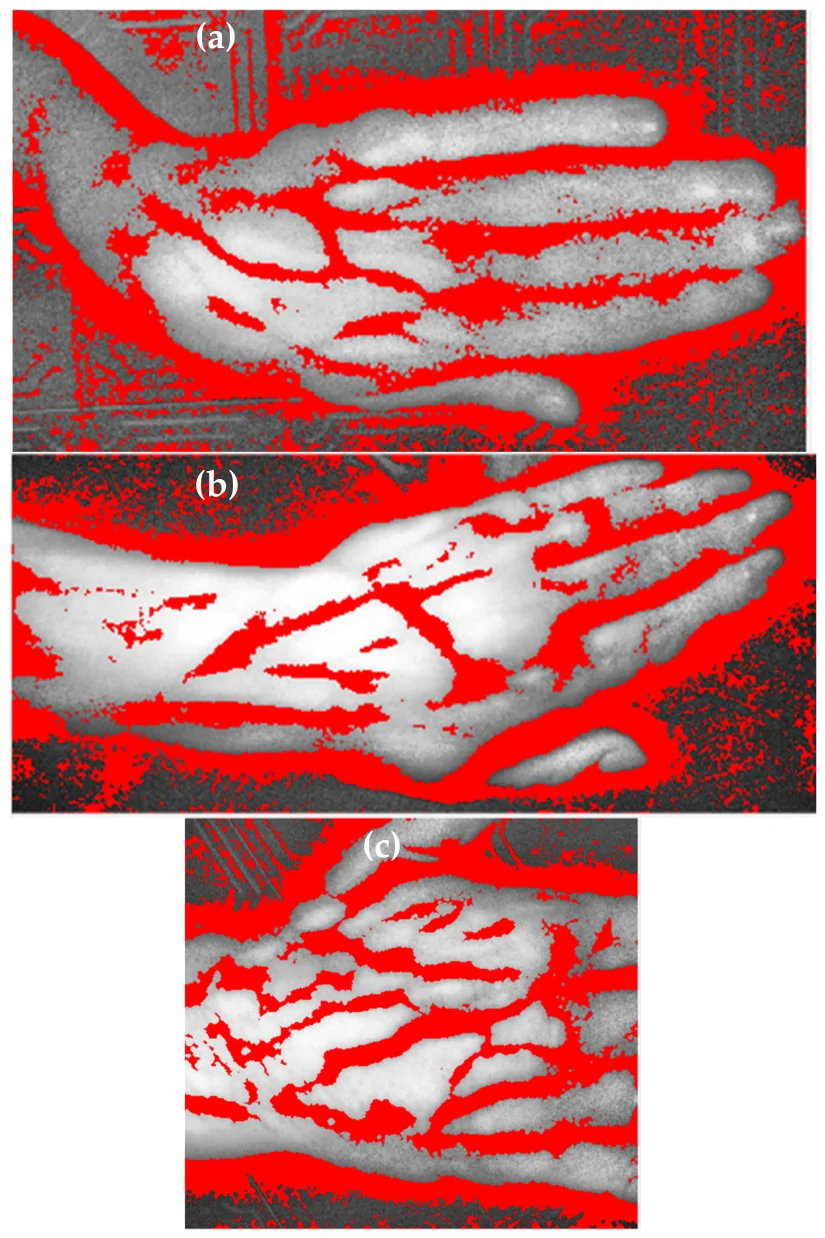
Processed near-infrared images of the dorsal aspect of the hand with the segmented vein mask overlaid in red: (**a**) Male subject, 24 years old; (**b**) Female subject, 48 years old; and (**c**) Male subject, 49 years old.

In the dorsal area of the hand, from the analysis of the equalized images, Figure 17, and the images to which the mask was applied, Figure 18, subject c, a 49-year-old male, showed the most prominent and numerous dorsal metacarpal veins, but in subject a, the venous arch of the dorsal metacarpals is much more pronounced.

**Figure 19:**
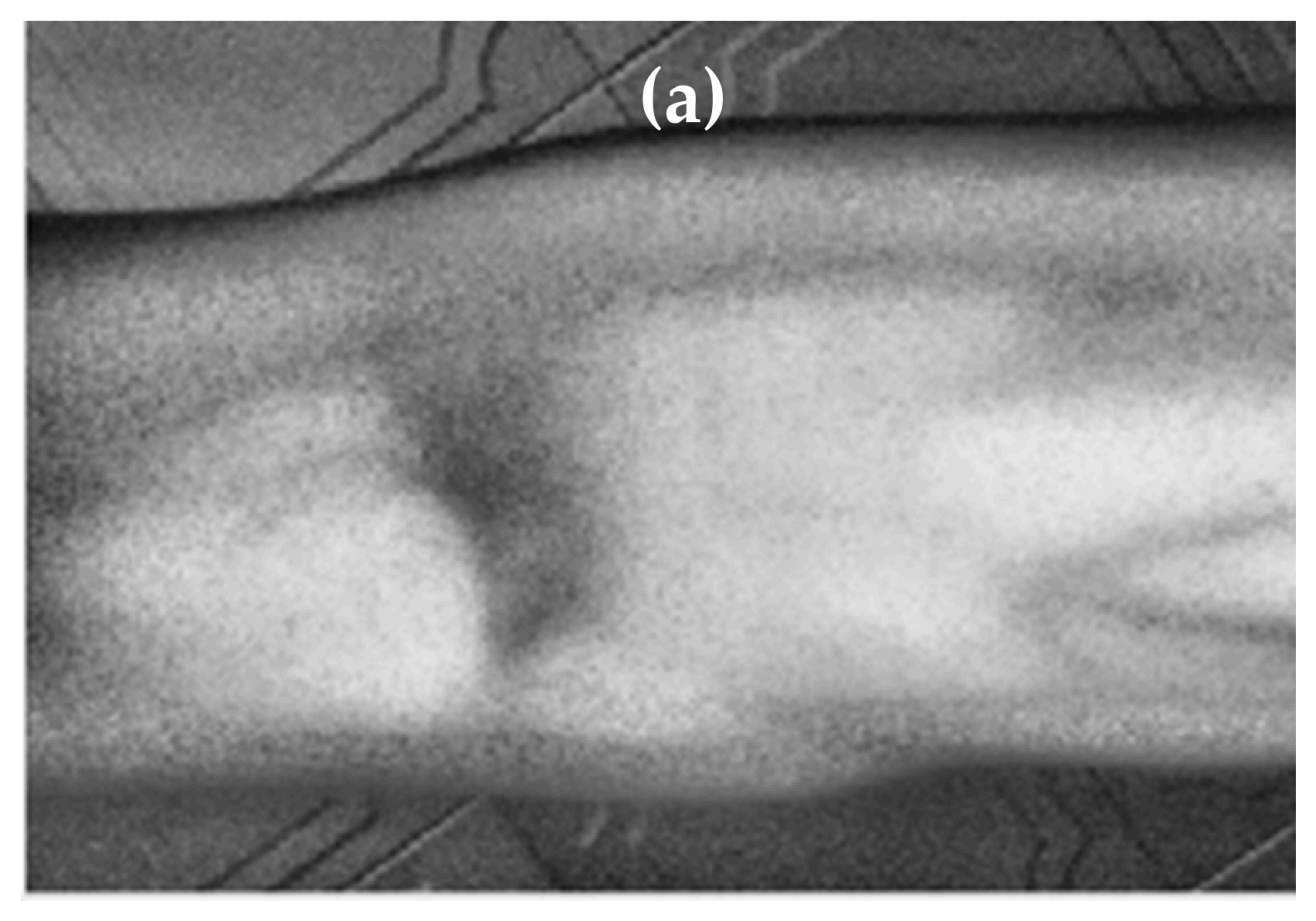

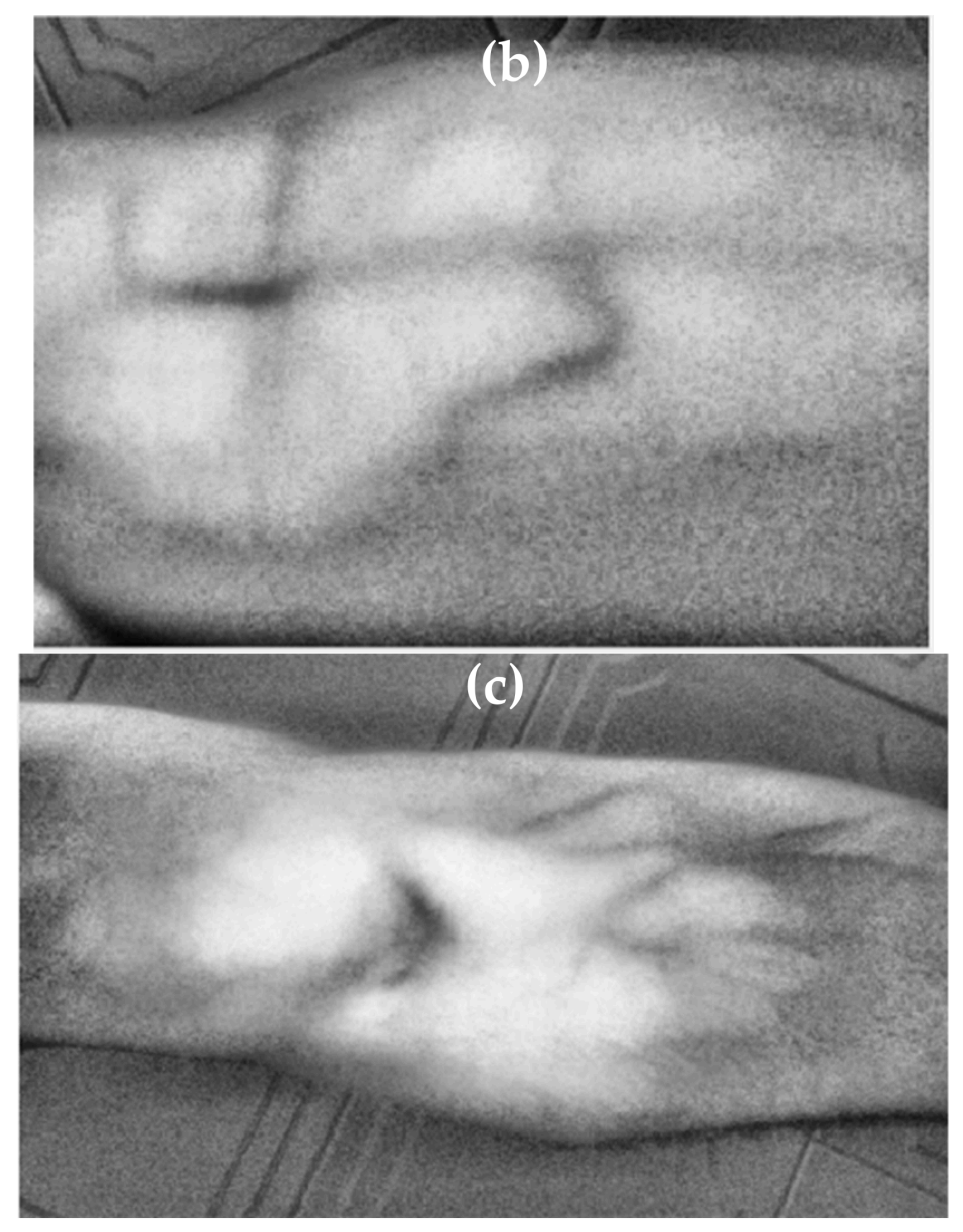
Near-infrared images of the antecubital fossa after adaptive histogram equalization: (**a**) Male subject, 24 years old; (**b**) Female subject, 48 years old; and (**c**) Male subject, 49 years old.

**Figure 20:**
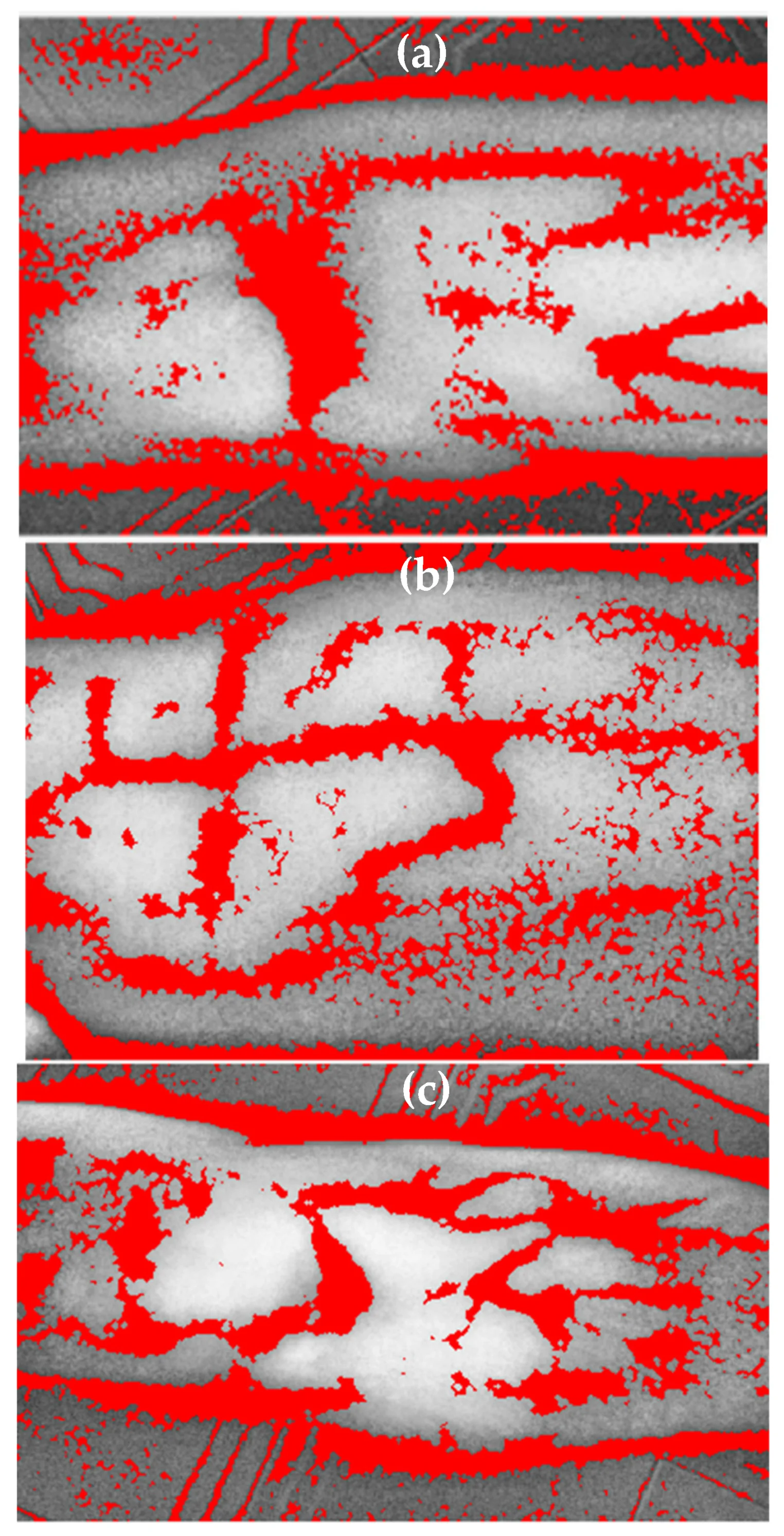
Processed near-infrared images of the antecubital fossa with the segmented vein mask overlaid in red: (**a**) Male subject, 24 years old; (**b**) Female subject, 48 years old; and (**c**) Male subject, 49 years old.

Finally, for the antecubital fossa, in the equalized images, Figure 19, and those to which the mask was applied, Figure 20, subject b, female, aged 48, showed the most clearly delineated median cubital vein.

**Figure 21:**
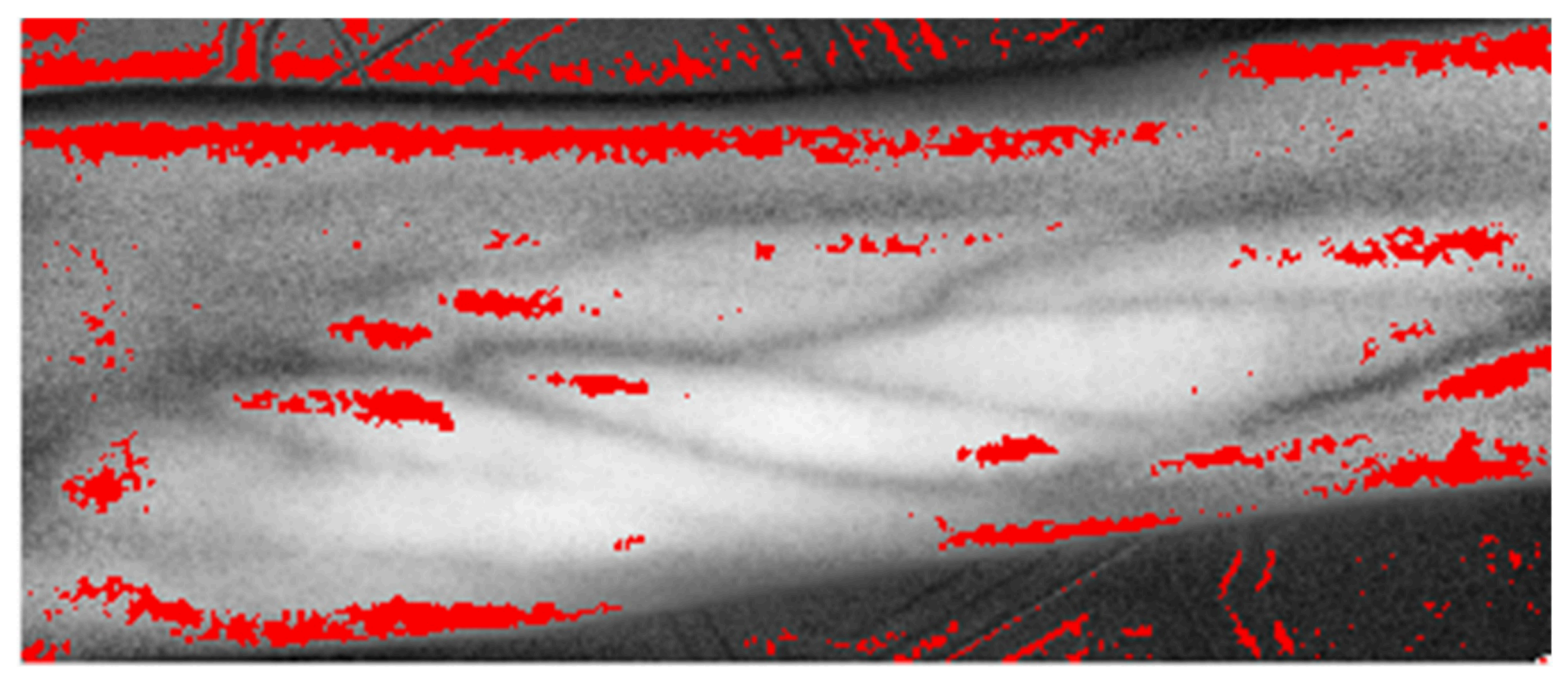
Initial vein-segmentation result for the ventral aspect of the forearm, showing incorrect application of the vein mask in red to adjacent non-vascular regions. The image was obtained using an adaptive threshold sensitivity value of 0.5 before optimization of the image-processing parameters.

The nine analyzed images were considered image-level technical observations obtained from three participants and were not treated as nine statistically independent biological samples. Three anatomical regions were examined for each participant, creating a repeated-measures structure. For this reason, the quantitative results are reported descriptively, and no inferential statistical claims were made. All these images were selected from a large set of tests performed. Also, during the first test, the program created underwent changes because the initial results were erroneous, Figure 21, the mask that was to be applied to highlight the veins being applied to surfaces adjacent to them. After checking each step individually, it was found that the initial adaptive threshold level was too low, at 0.5, and the solution was to increase the threshold to 0.6. The second error was at the image binarization level, as the resulting image had to be inverted in order to correctly outline the veins, as previously mentioned.

To complement the visual evaluation of the acquired vein images, a quantitative assessment was performed using two image-quality metrics: vein-to-background contrast and contrast-to-noise ratio (CNR). These metrics were calculated for the nine acquired images, corresponding to three anatomical zones examined in each of the three participants. The purpose of this analysis was not to provide clinical validation, but to determine objectively whether the proposed image-processing pipeline improved the distinction between superficial veins and surrounding tissue under the tested acquisition conditions.

For each image, regions of interest were selected from the visible vein area and from the adjacent background tissue. The same regions of interest were used for the unprocessed and processed images, to allow a direct comparison. The vein-to-background contrast (3) was calculated using the mean pixel intensity of the vein region and the surrounding background region, according to the following expression:

(3) $$\mathrm{Contrast}\;=\;\frac{\left|\;I_{\mathrm{background}}-\;I_{\mathrm{vein}}\right|}{I_{\mathrm{background}}},$$

where I_background_ represents the mean pixel intensity of the selected background region and I_vein_ represents the mean pixel intensity of the selected vein region. The contrast-to-noise ratio (4) was calculated to evaluate the difference between the vein and background regions while also considering the variability of pixel intensities inside these regions:

(4) $$\mathrm{CNR}\;=\;\frac{\left|\;{\mathrm{mean}}_{\mathrm{background}}\;-\;{\mathrm{mean}}_{\mathrm{vein}}\right|}{\sqrt{{{\mathrm{std}}_{\mathrm{background}}}^{2}+{{\mathrm{std}}_{\mathrm{vein}}}^{2}}}$$

where mean_background_ and mean_vein_ are the mean pixel intensities of the background and vein regions, while std_background_ and std_vein_ are their corresponding standard deviations.

The resulting contrast and CNR values are presented in Table 3:

**Table 3:** Quantitative assessment of vein-to-background contrast and contrast-to-noise ratio before and after image processing.

Participant	Zone/Anatomical Region	Contrast Before Processing	Contrast After Processing	Contrast Change (%)	CNR Before Processing	CNR After Processing	CNR Change (%)
Male 24	Ventral area of the forearm	0.10337	0.2517	143.49	0.31579	0.90444	186.41
	Dorsal area of the hand	0.13816	0.29867	116.18	0.33512	0.81639	143.61
	Antecubital fossa	0.16666	0.31773	90.653	0.43299	0.98692	127.93
Female 48	Ventral area of the forearm	0.094971	0.16461	73.321	0.94745	1.1275	19.004
	Dorsal area of the hand	0.33091	0.41277	24.738	0.59115	0.87482	47.986
	Antecubital fossa	0.090471	0.25693	183.99	0.41362	1.2518	202.65
Male 49	Ventral area of the forearm	0.13018	0.29085	123.41	0.47771	1.0946	129.14
	Dorsal area of the hand	0.19712	0.35195	78.544	0.52099	1.0554	102.57
	Antecubital fossa	0.095285	0.27275	186.25	0.30126	0.9537	216.57

To avoid relying only on relative percentage changes, the absolute differences between processed and unprocessed images are reported in Table 4. Across the analyzed images, all anatomical regions showed an increase in both contrast and CNR after processing. The absolute contrast difference ranged from 0.06964 to 0.17747, while the absolute CNR difference ranged from 0.18005 to 0.83818.

**Table 4:** Absolute differences in contrast and CNR after image processing.

Participant	Zone/Anatomical Region	Absolute Contrast Difference	Absolute Contrast Difference
Male 24	Ventral area of the forearm	0.14833	0.58865
	Dorsal area of the hand	0.16051	0.48127
	Antecubital fossa	0.15107	0.55393
Female 48	Ventral area of the forearm	0.06964	0.18005
	Dorsal area of the hand	0.08186	0.28367
	Antecubital fossa	0.16646	0.83818
Male 49	Ventral area of the forearm	0.16067	0.61689
	Dorsal area of the hand	0.15483	0.53441
	Antecubital fossa	0.17747	0.65244

Participant-level mean values are summarized in Table 5, and the overall descriptive results are presented in Table 6.

## 5. Discussion

The quantitative assessment in Table 3 showed that the proposed image-processing method improved both vein-to-background contrast and CNR in all nine analyzed images. Overall, contrast increased from a mean value of 0.14968 before processing to 0.29088 after processing, corresponding to an average improvement of approximately 113.40%. CNR increased from a mean value of 0.48179 to 1.00729, corresponding to an average improvement of approximately 130.65%. These results support the visual observation that the processed images provided a clearer separation between the superficial veins and the surrounding tissue.

Differences were observed between participants and anatomical zones. For Male 24 participant, contrast increased in all three zones, with improvements ranging from 90.653% in Antecubital fossa to 143.49% in Ventral area of the forearm. CNR also increased consistently, from 0.31579 to 0.90444 in Ventral area of the forearm, from 0.33512 to 0.81639 in Dorsal area of the hand, and from 0.43299 to 0.98692 in Antecubital fossa. This indicates that, for this participant, the processing method produced stable improvement across the regions examined, although the relative gain was higher in the areas with lower initial contrast. For Female 48 participant, the results were more variable. Ventral area of the forearm showed a moderate contrast increase, from 0.094971 to 0.16461, while CNR increased only from 0.94745 to 1.1275, corresponding to the smallest CNR improvement in the dataset, 19.004%. Dorsal area of the hand had the highest initial contrast value among all measurements, 0.33091, and increased to 0.41277 after processing, giving a lower relative improvement of 24.738%. This smaller percentage increase is expected because the initial contrast was already comparatively high. In contrast, Antecubital fossa showed a much stronger enhancement, with contrast increasing from 0.090471 to 0.25693 and CNR increasing from 0.41362 to 1.2518. These findings suggest that the processing pipeline had the greatest effect in regions where the initial vein-to-background contrast was low, but the vascular pattern was still present in the raw image. Male 49 participant also showed consistent improvement after processing. Contrast increased from 0.13018 to 0.29085 in Ventral area of the forearm, from 0.19712 to 0.35195 in Dorsal area of the hand, and from 0.095285 to 0.27275 in Antecubital fossa. The corresponding CNR values increased from 0.47771 to 1.0946, from 0.52099 to 1.0554, and from 0.30126 to 0.9537, respectively. The largest relative improvements for this participant were observed in Antecubital fossa, where contrast increased by 186.25% and CNR by 216.57%. This again indicates that the method was particularly effective when applied to images with lower initial contrast.

To complement the relative percentage changes, absolute differences were also analyzed. This was important because percentage increases can be influenced by the initial value of each image. When the baseline contrast or CNR is low, even a moderate numerical increase can produce a large relative percentage change. For this reason, the absolute contrast and CNR differences were included to describe the direct numerical effect of the processing pipeline. Across the analyzed images, all anatomical regions showed positive absolute differences for both contrast and CNR. The absolute contrast difference ranged from 0.06964 in Female 48 participant, Ventral area of the forearm, to 0.17747 in Male 49 participant, Antecubital fossa. The absolute CNR difference ranged from 0.18005 in Female 48 participant, Ventral area of the forearm, to 0.83818 in Female 48 participant, Antecubital fossa. These values show that the processing pipeline improved the separation between the selected vein and background regions in all image pairs, although the magnitude of improvement varied between participants and anatomical regions.

At zone level, the antecubital fossa showed the highest mean absolute improvement, with a mean contrast difference of 0.16500 and a mean CNR difference of 0.68152. This suggests that the processing pipeline was particularly effective in this region under the tested acquisition conditions. A possible explanation is that the raw images from this region contained visible vascular information, but with relatively low initial contrast, allowing the enhancement steps to produce a stronger measurable effect. In comparison, Dorsal area of the hand showed a lower mean absolute contrast difference of 0.13240 and a mean absolute CNR difference of 0.43312. This may be related to better initial image quality in some Dorsal area of the hand acquisitions, where the veins were already more distinguishable before processing and therefore allowed a smaller increase after enhancement. The participant-level summary provides another view of the enhancement effect. All three participants showed increased mean contrast and CNR after processing. Male 49 participant had the highest mean absolute contrast difference, 0.16432, and the highest mean absolute CNR difference, 0.60125. Male 24 participant also showed consistent improvement, with a mean absolute contrast difference of 0.15330 and a mean absolute CNR difference of 0.54128. Female 48 participant showed a lower mean absolute contrast difference of 0.10599 and a lower mean absolute CNR difference of 0.43397, although the processed values were still higher than the corresponding unprocessed values. These differences may be related to variation in initial vein visibility, local tissue thickness, vein depth, illumination uniformity, and camera positioning.

The overall descriptive summary showed that mean vein-to-background contrast increased from 0.14968 ± 0.07705 before processing to 0.29088 ± 0.06922 after processing, corresponding to a mean absolute increase of 0.14120. Mean CNR increased from 0.48179 ± 0.19970 to 1.00729 ± 0.13785, corresponding to a mean absolute increase of 0.52550. The lower standard deviation after processing suggests that the processed images may have produced more uniform contrast and CNR values across the analyzed dataset. This observation should be interpreted cautiously, however, because the dataset included only three participants and three anatomical regions per participant.

Taken together, these findings support the role of the proposed processing pipeline as an image-enhancement method for superficial vein visualization under the tested acquisition conditions. The nine analyzed images were obtained from three participants and therefore do not represent nine independent biological samples. Accordingly, the results should be interpreted as descriptive technical evidence rather than as evidence of generalizable performance. The increase in contrast and CNR indicate improved objective image-quality metric after processing. The increase in both contrast and CNR supports the feasibility of using this low-cost approach for enhancing vascular structures under controlled acquisition conditions. The observed differences between participants and anatomical regions also provide useful information about the factors that may influence system performance, such as vein depth, local tissue characteristics, illumination uniformity, and initial image quality. Therefore, the present study represents an important technical development stage and provides a quantitative basis for further optimization. The quantitative analysis based on contrast and CNR evaluates image enhancement and visibility improvement, not anatomical segmentation accuracy. Since no expert-annotated ground truth or independent reference method, such as Doppler ultrasound, was used, the generated binary masks should be interpreted as image-processing outputs that highlight vein-like structures in the NIR images. They should not be considered validated anatomical segmentations. Future work should compare the generated masks with expert annotations or reference imaging and report segmentation metrics such as Dice coefficient, IoU, sensitivity, specificity, and precision.

## 6. Conclusions

This paper aimed to develop a low-cost NIR-based prototype that combines accessible hardware with a reproducible image-processing method for enhancing superficial vein visibility consisting of a camera without an infrared light filter and an infrared LED lighting module, to visualize superficial veins in a non-invasive manner. Based on the knowledge acquired from NIR spectroscopy and optics, we know that when the surface of the human body is illuminated with our infrared LED module, superficial veins can produce sufficient optical contrast relative to adjacent tissue to become more visible in NIR images. The visibility of superficial veins in NIR images results from differences in the wavelength-dependent absorption and scattering properties of blood and surrounding tissue, with hemoglobin contributing to the optical contrast observed between vascular structures and adjacent tissue. These veins will be captured by the special camera used, through the development plate. The images obtained will then be transferred to a computer using a memory stick, where, to improve image clarity and highlight the veins, they will be processed using a MATLAB-based program we have created. The processing sequence, which included median filtering, histogram-based contrast enhancement, adaptive thresholding, binarization, image inversion, erosion, masking, and perimeter extraction, allowed the superficial vascular pattern to be more clearly distinguished in the acquired images.

The quantitative assessment supported the visual observations obtained during image analysis. Across the nine images acquired from three participants and three anatomical regions per participant, the mean vein-to-background contrast increased from 0.14968 ± 0.07705 before processing to 0.29088 ± 0.06922 after processing, corresponding to a mean relative improvement of 113.40% and to a mean absolute increase of 0.14120. Similarly, the mean contrast-to-noise ratio increased from 0.48179 ± 0.19970 to 1.00729 ± 0.13785, corresponding to a mean relative improvement of 130.65% and to a mean absolute increase of 0.52550. These results indicate that the proposed processing pipeline improved the objective visibility of superficial veins under the tested acquisition conditions. The degree of improvement varied between participants and anatomical regions, suggesting that image quality and processing performance are influenced by factors such as vein depth, local tissue thickness, skin characteristics, illumination uniformity, camera positioning, and the initial visibility of the vascular structures in the raw near-infrared images. This variability is relevant because it shows that the system’s performance depends not only on the processing algorithm, but also on the quality of the acquired image and the anatomical characteristics of the examined region.

The present work represents an initial technical foundation feasibility study and provides a quantitative basis for the continued development of the proposed system. The results show that a low-cost near-infrared imaging setup, combined with a reproducible MATLAB-based processing pipeline, can enhance the visibility of superficial veins under controlled acquisition conditions. The improvements observed in contrast and CNR support the potential of this approach as an accessible tool for vein visualization.

Looking ahead, as technology continues to advance, it is anticipated that the NIR vein locator device developed in this work will undergo further improvements and refinements. Future iterations will build on these findings by expanding the participant group, including different age categories, skin types, and vascular conditions, and by evaluating the prototype in more clinically relevant venipuncture scenarios. Further optimization of illumination, camera positioning, and user interfaces to ease and simplify the work of filtering, real-time processing, and segmentation accuracy may increase the robustness of the system and support its development toward practical biomedical applications. Another future direction for the development of the device is to establish a real-time connection with the filtering program, which will further reduce working time, and to project the veins onto the patient’s skin surface for puncture accuracy, which is the main purpose of this device. A future version of the prototype should include a low-cost enclosure, potentially manufactured by 3D printing, to standardize the distance between the camera and skin, the angle of acquisition, and the position of the infrared LED module. Such an enclosure would reduce operator-dependent variability and improve the repeatability of image acquisition across participants and anatomical regions.

## Figures and Tables

**Table 1:** Comparison of Vein Imaging Technologies.

Imaging Modality	Hardware	Processing Method	Strengths	Limitations
Reflected Near-Infrared (NIR) [3]	NIR light source, IR-sensitive camera, integrated display/projector.	Proprietary real-time image mapping.	Handheld, portable, provides an immediate map on skin.	Commercial devices get to an extremely high cost.
Ultrasound (Sound Waves) [6,7]	Transducer probe, processing unit, and monitor.	Doppler and B-mode signal processing.	Determines exact depth, diameter, and blood flow velocity; differentiates arteries and veins.	Requires high operator expertise and may be expensive/less portable.
Visible Light Transillumination [8]	High-intensity LEDs (often orange/red/white) in C-shaped rings.	Direct visual observation by the practitioner.	Very portable and cost-effective; good for superficial veins in infants.	Restricted to thin tissue areas; high light intensity can potentially cause heat/burns.
Laser-based Infrared Imaging [4,5]	Nd:YAG or diode lasers, CCD/InGaAs camera, filters, and diffusers.	Contrast enhancement via real-time video signal processing.	Highest monochromaticity and resolution; can monitor therapy margins.	Laser safety risks (e.g., retinal burns); high system complexity.
Academic AR/Projector Prototypes [2,3,8]	Raspberry Pi, NoIR Camera, Pico-projector, IR LED array.	Median filtering and Adaptive Histogram Equalization.	Offers favorable ergonomics among optical systems because the user looks at the patient, not away to a separate screen.	Does not measure vein depth, may not improve first-stick success in all groups, higher cost

**Table 2:** Wavelength, wave number, and relative intensity value of the bands due to the fundamental, first, second, and third overtones of CH for the stretching mode of chloroform.

	Band Position [nm]	Band Position [cm^−1^]	Intensity [cm^2^mol^−1^]
υ	3290	3040	25,000
2 υ	1693	5907	1620
3 υ	1154	8666	48
4 υ	882	11,338	1.7
5 υ	724	13,831	0.15

**Table 5:** Participant-level summary of contrast and CNR before and after processing.

Participant	Mean Contrast Before	Mean Contrast After	Mean Absolute Contrast Difference	Mean Contrast Change (%)	Mean CNR Before	Mean CNR After	Mean Absolute CNR Difference	Mean CNR Change (%)
Male 24	0.13606	0.28937	0.15330	116.774	0.36130	0.90258	0.54128	152.650
Female 48	0.17212	0.27810	0.10599	94.016	0.65074	1.08471	0.43397	89.880
Male 49	0.14086	0.30518	0.16432	129.401	0.43332	1.03457	0.60125	149.427

**Table 6:** Overall descriptive summary of contrast and CNR.

Metric	Before Processing, Mean ± SD	After Processing, Mean ± SD	Mean Absolute Difference	Mean Relative Change (%)
Contrast	0.14968 ± 0.07705	0.29088 ± 0.06922	0.14120	113.397
CNR	0.48179 ± 0.19970	1.00729 ± 0.13785	0.52550	130.652

Mean contrast increased from 0.14968 ± 0.07705 before processing to 0.29088 ± 0.06922 after processing, while mean CNR increased from 0.48179 ± 0.19970 to 1.00729 ± 0.13785.

## Data Availability

The raw and processed near-infrared images analyzed in this study are not publicly available due to participant confidentiality and privacy considerations. Anonymized quantitative measurements, and where ethically permissible, anonymized image data, may be made available by the corresponding author upon reasonable request.

## Abbreviations

The following abbreviations are used in this manuscript:

NIR	Near-Infrared
IR	Infrared
AR	Augmented Reality
CV	Cardiovascular
IV	Intravenous
VAT	Vein assessment tool
MR	Magnetic Resonance
Hb	Hemoglobin
HbO2	Oxygenated hemoglobin
Nd:YAG	Neodymium-doped Yttrium Aluminum Garnet
InGaAs	Indium Gallium Arsenide
